# Antibody-Mediated Porcine Reproductive and Respiratory Syndrome Virus Infection Downregulates the Production of Interferon-α and Tumor Necrosis Factor-α in Porcine Alveolar Macrophages via Fc Gamma Receptor I and III

**DOI:** 10.3390/v12020187

**Published:** 2020-02-08

**Authors:** Liujun Zhang, Wen Li, Yangyang Sun, Linghao Kong, Pengli Xu, Pingan Xia, Gaiping Zhang

**Affiliations:** College of Animal Science and Veterinary Medicine, Henan Agricultural University, Zhengzhou 450002, China; lanlan@henau.edu.cn (L.Z.); lw839466399@163.com (W.L.); syy163yx@126.com (Y.S.); klh1102@163.com (L.K.); xpl129012@163.com (P.X.); zhanggaiping2003@163.com (G.Z.)

**Keywords:** FcγRI, FcγRIII, PRRSV, ADE, IFN-α, TNF-α

## Abstract

Antibody-dependent enhancement (ADE) contributes to the pathogenesis of porcine reproductive and respiratory syndrome virus (PRRSV)-persistent infection. However, the mechanisms of PRRSV-ADE infection are still confusing. A clear understanding of the event upon virus infection by the ADE pathway has become crucial for developing efficient intervention of the PRRSV infection. In this study, an ADE assay showed that PRRSV-ADE infection in porcine alveolar macrophages (AMs) significantly decreased the production of interferon-α (IFN-α) and tumor necrosis factor-α (TNF-α), and significantly increased the production of interleukine-10 (IL-10). A gene knockdown assay based on small interfering RNA (siRNA) showed that both Fc gamma receptor I (FcγRI) and FcγRIII in porcine AMs were involved in PRRSV-ADE infection. An activation assay showed that specific activation of FcγRI or FcγRIII in porcine AMs during PRRSV infection not only significantly decreased the production of IFN-α and TNF-α, but also significantly increased the production of IL-10 and significantly facilitated PRRSV replication. In conclusion, our studies suggested that ADE downregulated the production of IFN-α and TNF-α in porcine AMs maybe via FcγRI and FcγRIII, thereby leading to enhanced PRRSV infection.

## 1. Introduction

Receptors for the Fc portion (FcγRs) of immunoglobulin G (IgG) belong to the Ig receptor superfamily and are expressed on the surface of most immune cells [1]. FcγRs play important roles in the innate and adaptive immune responses by triggering pleiotropic biological functions, such as release of inflammatory mediators, regulation of cellular activation and differentiation, controlling of peripheral tolerance, processing and presentation of antigens, phagocytosis of microorganisms, endocytosis of immune complexes, antibody-dependent cellular cytotoxicity and antibody protection against viral infection [2,3,4,5]. Functionally, FcγRs from mammalian species are categorized into the activating (FcγRI, FcγRIIa/IIc, FcγRIII and FcγRIV) and inhibitory (FcγRIIb) receptors [6,7]. The two different types of receptors display coordinate and opposing roles in the immune system. FcγRs are also distinguished by their varying affinity for IgG. FcγRI exhibits a high affinity for IgG and is capable of binding monomeric IgG [1]. FcγRII and FcγRIII show a low affinity for monomeric IgG and can only interact effectively with multimeric immune complexes [8]. FcγRIV is able to bind to IgG2a and IgG2b with an intermediate affinity [9]. With the exception of B cells and natural killer cells, which exclusively express FcγRIIb and FcγRIII respectively, one hallmark of the FcγR family is the paired expression of activating and inhibitory receptors on the same cells [10,11]. Therefore, the cellular activation and the regulation of cellular effector functions depend on the simultaneous initiation of the activating and inhibitory receptor signals.

Porcine reproductive and respiratory syndrome (PRRS) caused by the PRRSV (V, virus) is one of the most important infectious diseases influencing the pig industry worldwide [12]. PRRSV is an enveloped, single-stranded, positive-sense RNA virus belonging to the family *Arteriviridae* in the order *Nidovirales* [12,13]. This virus is highly host- and tissue-restricted to swine cells of the monocyte/macrophage lineage, preferentially infecting porcine AMs [14,15,16]. The infected swines produce a rapid humoral immune response, but the sub- or non-neutralizing antibody specific against PRRSV may contribute to the development of PRRS. Enhanced infection and replication of PRRSV in the presence of the antibody specific for the virus has been demonstrated in vivo and in vitro [17,18], a phenomenon known as antibody-dependent enhancement (ADE) that promotes the attachment and internalization of the virus into its host cells by Fc receptor-mediated endocytosis [19,20,21,22]. In addition, ADE has been implicated as a major obstacle to the development of efficacious vaccines for many viruses, including PRRSV [20,23].Nevertheless, there is also a report that there is no evidence for a role for antibodies during vaccination-induced enhancement of PRRSV [24].

Porcine-activating FcγRs, including FcγRI and FcγRIII, have been cloned and characterized [25,26,27]. Previous studies have shown that FcγRI or FcγRIII ligation in porcine AMs suppresses the innate antiviral immune response [28,29]. But, their roles in innate antiviral immune response to PRRSV infection by the ADE pathway have not yet been investigated. In this study, we report the effects of FcγRI and FcγRIII on innate antiviral immune response to PRRSV-ADE infection in porcine AMs.

## 2. Materials and Methods

### 2.1. Cells and Virus

Porcine AMs were isolated from PRRSV-negative pigs of four to six-weeks-old and cultured in Roswell Park Memorial Institute medium (HyClone, Logan, UT, USA) containing 10% fetal bovine serum (FBS) (HyClone). Marc-145 cells were highly permissive for PRRSV infection and were cultured in Dulbecco's modified Eagle's medium (HyClone) containing 10% FBS. North American-type PRRSV strain HeN-3 (GenBank ID: FJ237420) was isolated in Marc-145 cells. PRRSV titers were determined in Marc-145 cells for the 50% tissue culture infectious dose (TCID_50_), as specified by the Reed–Muench method.

### 2.2. Antibodies

The polyclonal antibody (pAb) specific for PRRSV (Enzyme-linked immunosorbent assay (ELISA) antibody titer: 6400) was derived from pigs immunized with inactivated purified HeN-3 viral particles coupled with Freund’s Incomplete Adjuvant (Sigma-Aldrich, Saint Louis, MO, USA). The pAb specific for FcγRI (ELISA antibody titer: 12800) was derived from rabbits immunized with extracellular domain proteins of porcine FcγRI coupled with Freund’s Incomplete Adjuvant. The pAb specific for FcγRIII (ELISA antibody titer: 12800) was derived from rabbits immunized with extracellular domain proteins of porcine FcγRIII coupled with Freund’s Incomplete Adjuvant. The IgG specific for PRRSV or FcγRI or FcγRIII was purified from each pAb by ammonium sulphate precipitation and diethyl-aminoethanol chromatography. Porcine-negative IgG and rabbit-negative IgG were purified from the serum of PRRSV-negative piglets and the serum of healthy rabbits, respectively. Anti-phospho-IRF3 (Ser396) rabbit monoclonal antibody (mAb) was purchased from Invitrogen (NY, USA). Horseradish peroxidase (HRP)-linked anti-rabbit IgG antibody and anti-glyceraldehyde-3-phosphate dehydrogenase (GAPDH) rabbit mAb (HRP conjugate) were purchased from Cell Signaling Technology (Boston, MA, USA). Fluorescein isothiocyanate (FITC)-conjugated goat anti-rabbit IgG antibody was purchased from Proteintech Group (Wuhan, China).

### 2.3. Generation of Infectious PRRSV Immune Complexes (ICs)

The porcine IgG specific for PRRSV (850 μg/mL) was mixed with equal volumes of PRRSV suspensions (2000 TCID_50_/mL) at 37 °C for 1 h to form infectious virus ICs (PRRSV+ICs). Simultaneously, the porcine-negative IgG (850 μg/mL) was mixed with equal volumes of PRRSV suspensions (2000 TCID_50_/mL) at 37 °C for 1 h to form the negative control (PRRSV+PNI).

### 2.4. PRRSV-ADE Infection Assay in Porcine AMs

Porcine AMs were prepared in a 24-well plate (Corning, NY, USA) at a ratio of 5 × 10^5^ cells/well. The cells were incubated with 200 µL PRRSV suspensions (200 TCID_50_) or PRRSV+PNI, PRRSV+ICs or poly (I:C) (120 μg/mL) (Sigma-Aldrich) at 37 °C for 2 h. Then, the medium was changed to 500 µL fresh growth medium. Normal cells were used as a mock trial. The cells and culture supernatants were collected every 12 h post-infection for relative quantitative RT-PCR, ELISA or western blot. Simultaneously, PRRSV RNA copies in culture supernatants of infected cells were quantified by previously described real-time RT-PCR [30] and PRRSV titers in culture supernatants of infected cells were determined in Marc-145 cells by the Reed–Muench method.

### 2.5. RNA Interference Assay in Porcine AMs

Small interfering RNA (siRNA) targeting porcine FcγRI or FcγRIII and negative siRNA (shown in Table 1) were designed and synthesized by GenePharma (Shanghai, China) and then used for silencing the target genes. Briefly, porcine AMs were prepared in a 24-well plate at a ratio of 5 × 10^5^ cells/well and transfected with 20 pmol FcγRI siRNA, FcγRIII siRNA or negative siRNA by using lipofectamine 2000 (Invitrogen, NY, USA). The cells were collected at 24, 36, 48, 60 and 72 h post-transfection for relative quantitative RT-PCR or western blot or flow cytometry. Additionally, after 48 h transfection, the transfected cells were incubated with 200 µL PRRSV+ICs at 37 °C for 2 h. Then, the medium was changed to 500 µL fresh growth medium. The cells and culture supernatants were collected at 12 and 24 h post-infection for relative quantitative RT-PCR, ELISA or real-time RT-PCR or virus titration.

### 2.6. Activation Assay of FcγRI or FcγRIII in Porcine AMs

Porcine AMs were prepared in a 24-well plate at a ratio of 5 × 10^5^ cells/well. The cells were incubated with 200 µL anti-FcγRI IgG (300 μg/mL), anti-FcγRIII IgG (300 μg/mL) or rabbit-negative IgG (300 μg/mL) at 37 °C for 2 h. Then, the medium was changed to 500 µL fresh growth medium. Normal cells were used as a mock trial. The cells and culture supernatants were collected every 12 h post-treatment for relative quantitative RT-PCR, ELISA or western blot.

### 2.7. Effect of Activation of FcγRI or FcγRIII on Poly (I:C)-Induced Production of Cytokines in Porcine AMs

Porcine AMs were prepared in a 24-well plate at a ratio of 5 × 10^5^ cells/well. The cells were pretreated with 200 µL anti-FcγRI IgG (300 μg/mL), anti-FcγRIII IgG (300 μg/mL) or rabbit-negative IgG (300 μg/mL) at 37 °C for 2 h. The treated cells were incubated with 200 µL poly (I:C) (120 μg/mL) at 37 °C for 2 h. Then, the medium was changed to 500 µL fresh growth medium. The mock cells and poly (I:C)-stimulated cells served as controls. The cells and culture supernatants were collected every 12 h post-treatment for relative quantitative RT-PCR, ELISA or western blot.

### 2.8. Effect of Activation of FcγRI or FcγRIII on PRRSV-Induced Production of Cytokines in Porcine AMs

Porcine AMs were prepared in a 24-well plate at a ratio of 5 × 10^5^ cells/well. The cells were pretreated with 200 µL anti-FcγRI IgG (300 μg/mL), anti-FcγRIII IgG (300 μg/mL) or rabbit-negative IgG (300 μg/mL) at 37 °C for 2 h. The treated cells were incubated with 200 µL PRRSV suspensions containing 200 TCID_50_ at 37 °C for 2 h. Then, the medium was changed to 500 µL fresh growth medium. PRRSV-infected cells served as control. The cells and culture supernatants were collected every 12 h post-infection for relative quantitative RT-PCR, ELISA, western blot, real-time RT-PCR or virus titration.

### 2.9. RNA Extraction and Relative Quantitative RT-PCR

The extraction of total RNA from porcine AMs and the analysis of relative quantitative RT-PCR were performed as previously described [30]. The primers used for the relative quantitative RT-PCR are showed in Table 2. The target transcript was calculated with the difference (∆C_T_) between the C_T_ values of the target and the reference: ∆C_T_ = C_T_ (target) − C_T_ (reference). This was followed by transforming these values to absolute values by using the formula: comparative expression level = 2^−∆C^_T_.

### 2.10. ELISA Assay

Detections of IFN-α, TNF-α and IL-10 protein levels in each sample were done by using porcine IFN-α (Sigma-Aldrich), porcine TNF-α (Sigma-Aldrich) and porcine IL-10 (Invitrogen) ELISA Kits respectively, according to the manufacturer’s instructions. The protein concentrations of IFN-α, TNF-α and IL-10 were calculated on the basis of each standard curve that was obtained by using a known standard. All detections were performed in parallel.

### 2.11. Western Blot Assay

For the western blot experiment, the primary antibodies, anti-phospho-IRF3 (Ser396) rabbit mAb (1:1000 dilution), anti-FcγRI rabbit pAb (20 μg/mL) and anti-FcγRIII rabbit pAb (20 μg/mL) were used. Whole-cell lysates from porcine AMs were extracted. Similar amounts of protein from each extract were separated by 10% sodium dodecyl sulfate-polyacrylamide gel electrophoresis and transferred to polyvinylidenedifluoride (PVDF) membrane (Sigma-Aldrich). After blocking, the PVDF membranes were incubated with the indicated primary antibodies and the secondary HRP-linked anti-rabbit IgG antibody (1:3000 dilution). The anti-GAPDH rabbit mAb (HRP conjugate) (1:1000 dilution) was used to detect the reference protein. Then, the immunolabeled proteins were detected by using the ECL western blotting detection reagent (GE Healthcare, Boston, MA, USA).

### 2.12. Flow Cytometry Assay

Porcine AMs were collected and pretreated with anti-FcγRIIgG (100 μg/mL), anti-FcγRIIIIgG (100 μg/mL) or RNI (100 μg/mL) at 37 °C for 1 h. The treated cells were incubated with the secondary FITC-conjugated goat anti-rabbit IgG antibody (1:100 dilution) at 37 °C for 1 h. In every step, the cells were washed three times with phosphate buffer saline containing 3% FBS. Flow cytometry was performed by using the CytoFLEX flow cytometer (Beckman Coulter, Brea, California, USA).

### 2.13. Statistical Analysis

Data were expressed as mean ± standard error of mean from three independent experiments. Results were analyzed by the two-way analysis of variance (ANOVA) followed by the Bonferroni post-tests using the GraphPad Prism 5.0 software. The *p* values < 0.05 were considered to be statistically significant (*** *p* < 0.001, ** *p* < 0.01, * *p* < 0.05).

## 3. Results

### 3.1. Porcine IgG Specific for PRRSV Facilitates PRRSV Replication in Porcine AMs

**Figure 1:**
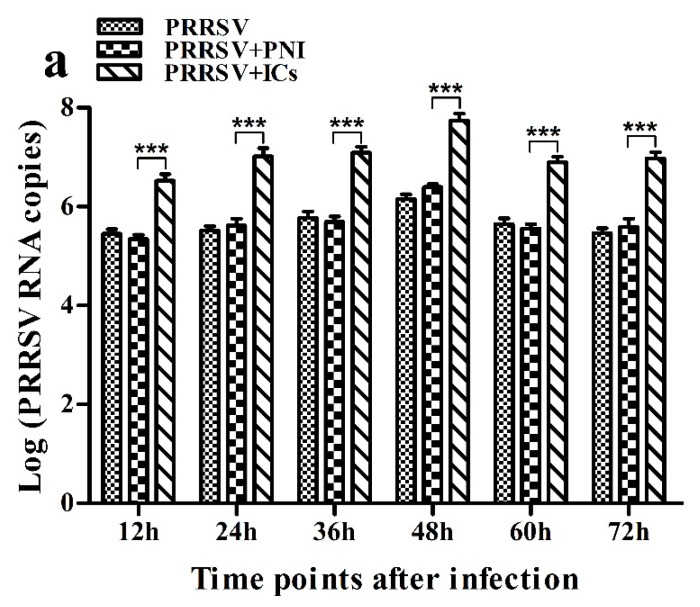

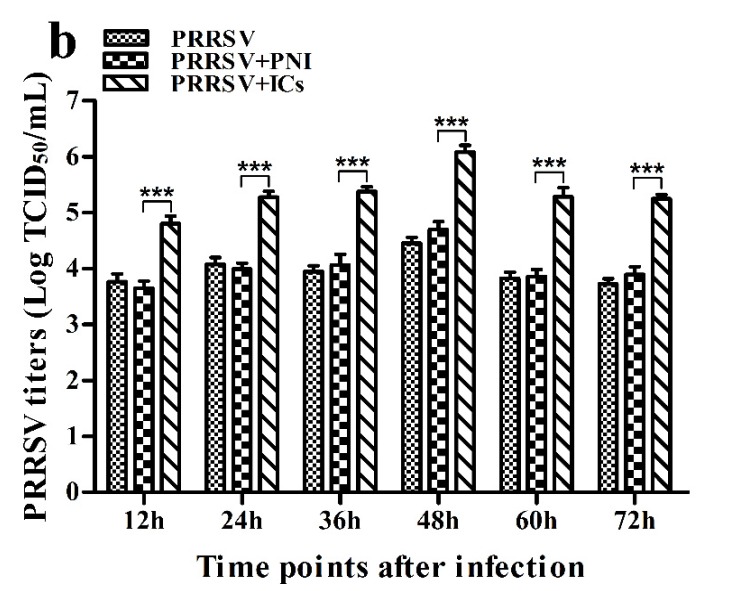
Effect of porcine IgG specific for PRRSV on PRRSV replication in porcine AMs. (**a**) Real-time RT-PCR analysis of PRRSV RNA copies in culture supernatants of cells infected with PRRSV, PRRSV+PNI or PRRSV+ICs. (**b**) PRRSV titers in culture supernatants of cells infected with PRRSV, PRRSV+PNI or PRRSV+ICs were measured in Marc-145 cells and expressed as TCID_50_/mL. *** *p* < 0.001. Note: PRRSV+PNI: PRRSV+porcine-negative IgG; PRRSV+ICs: PRRSV+porcine IgG against PRRSV.

As shown in Figure 1, both PRRSV RNA copies and its titers in culture supernatants of PRRSV+ICs-infected cells were significantly more than that in culture supernatants of PRRSV+PNI-infected cells at any time point post-infection, showing that PRRSV-ADE activity was present in porcine IgG against PRRSV.

### 3.2. PRRSV Infection Induces the Production of IFN-α and TNF-α in Porcine AMs

**Figure 2:**
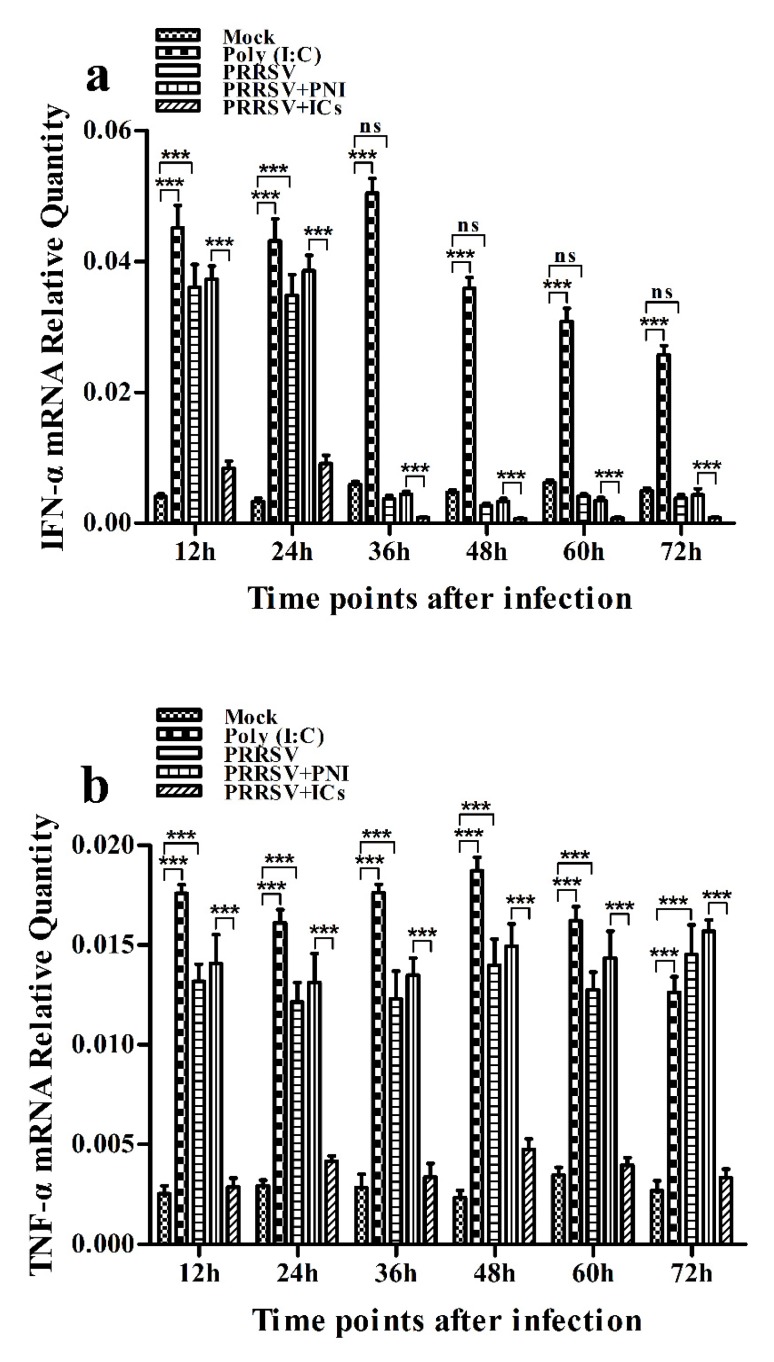

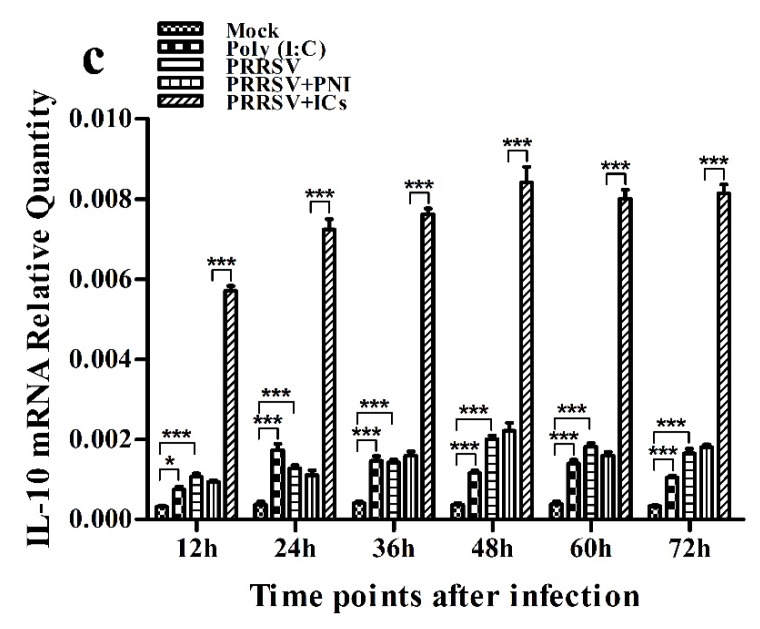
Effect of PRRSV infection or PRRSV-ADE infection on mRNA expression of cytokines in porcine AMs. Relative quantitative RT-PCR analysis of cytokine mRNA levels in mock cells, poly (I:C)-stimulated cells, PRRSV-infected cells, PRRSV+PNI-infected cells or PRRSV+ICs-infected cells. (**a**) IFN-α mRNA level, (**b**) TNF-α mRNA level, (**c**) IL-10 mRNA level. *** *p* < 0.001, * *p* < 0.05, ns: no significance. Note: PRRSV+PNI: PRRSV+porcine-negative IgG; PRRSV+ICs: PRRSV+porcine IgG against PRRSV; PRRSV-ADE: antibody-dependent enhancement of PRRSV.

**Figure 3:**
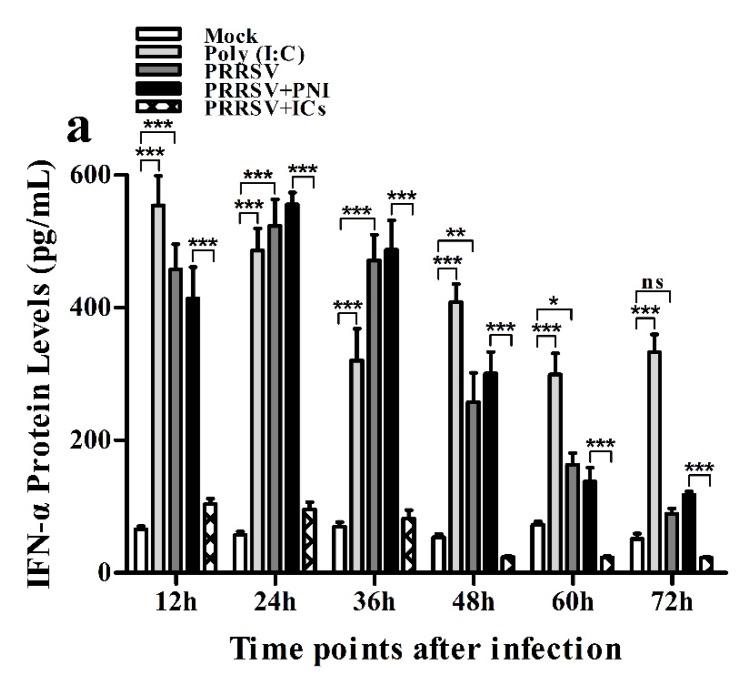

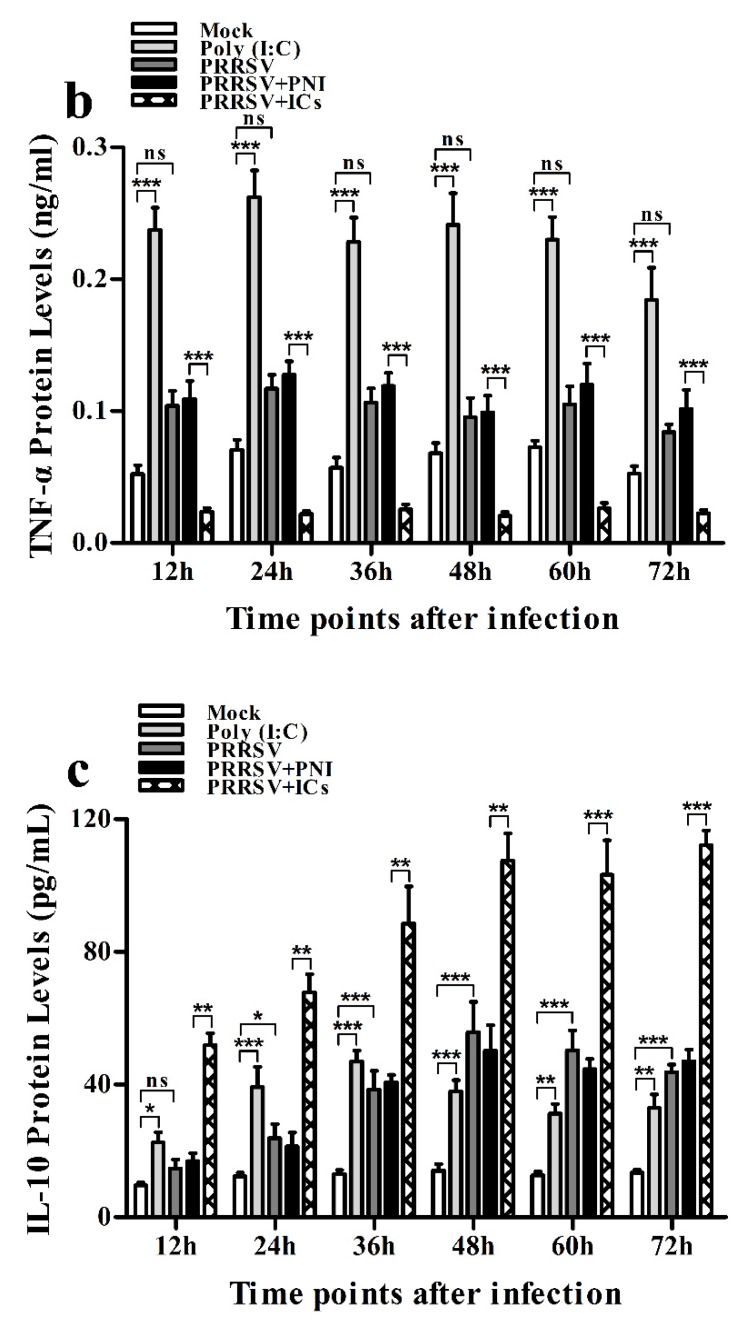
Effect of PRRSV infection or PRRSV-ADE infection on protein expression of cytokines in porcine AMs. Cytokine protein levels in culture supernatants of mock cells, poly (I:C)-stimulated cells, PRRSV-infected cells, PRRSV+PNI-infected cells or PRRSV+ICs-infected cells were detected by using ELISA Kits. (**a**) IFN-α protein level, (**b**) TNF-α protein level, (**c**) IL-10 protein level. *** *p* < 0.001, ** *p* < 0.01, * *p* < 0.05, ns: no significance. Note: PRRSV+PNI: PRRSV+porcine-negative IgG; PRRSV+ICs: PRRSV+porcine IgG against PRRSV; PRRSV-ADE: antibody-dependent enhancement of PRRSV.

**Figure 4:**
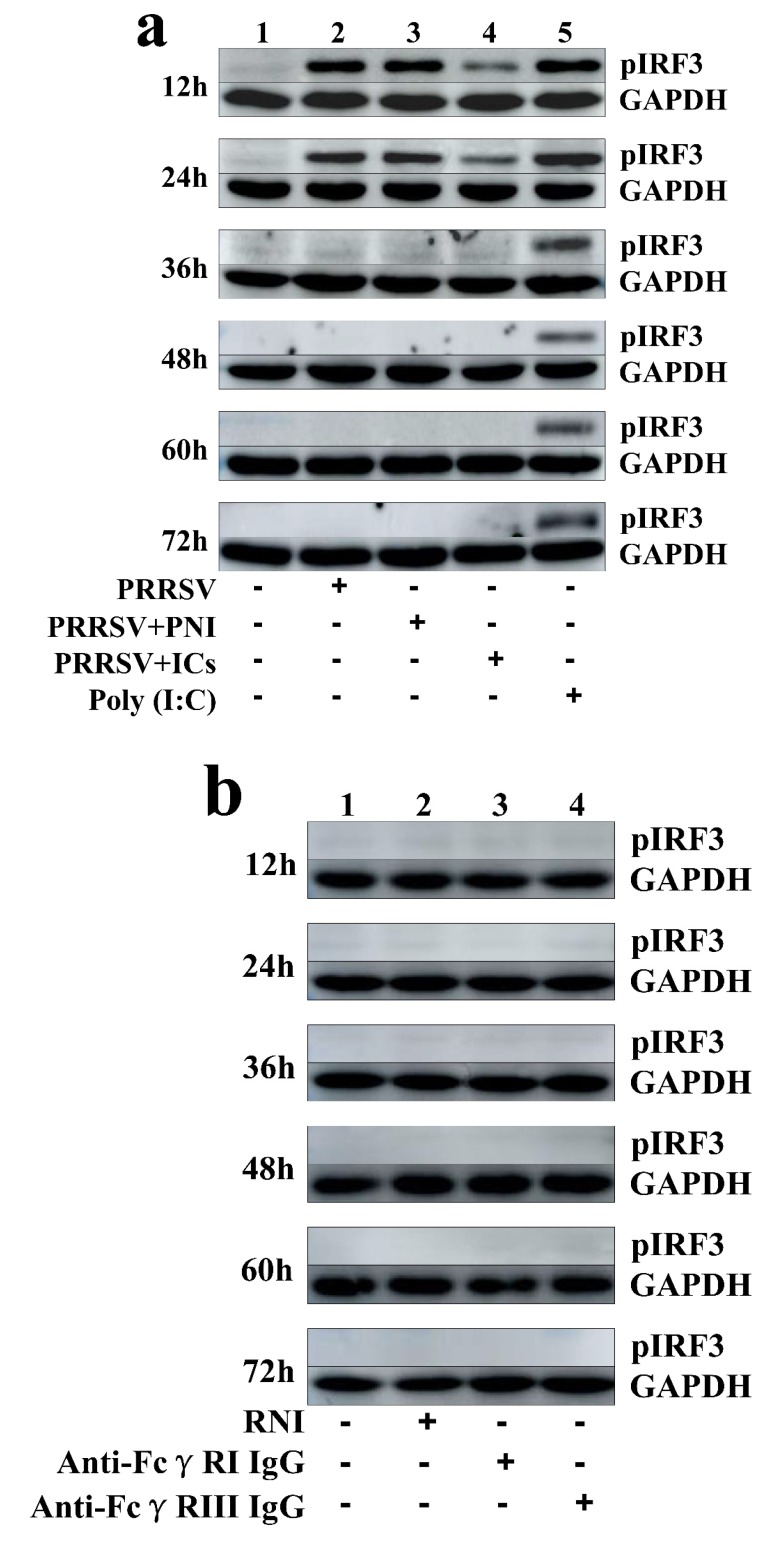

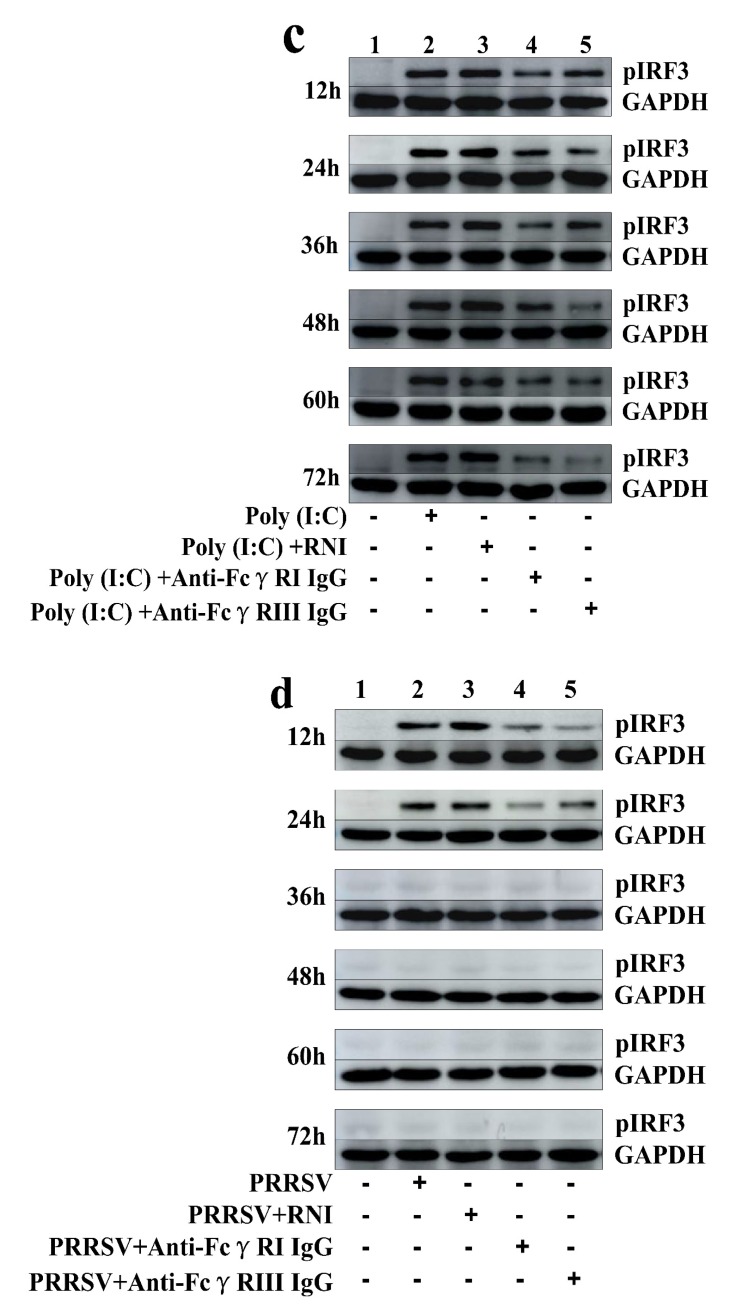
Immunoblot analysis of pIRF3 in porcine AMs. (**a**) Immunoblot analysis of pIRF3 in mock cells, poly (I:C)-stimulated cells, PRRSV-infected cells, PRRSV+PNI-infected cells or PRRSV+ICs-infected cells. (**b**) Immunoblot analysis of pIRF3 in cells treated with RNI, anti-FcγRI IgG or anti-FcγRIII IgG. (**c**) Immunoblot analysis of pIRF3 in poly (I:C)-stimulated cells pretreated with RNI, anti-FcγRI IgG or anti-FcγRIII IgG. (**d**) Immunoblot analysis of pIRF3 in PRRSV-infected cells pretreated with RNI, anti-FcγRI IgG or anti-FcγRIII IgG. The GAPDH served as a loading control. Note: PRRSV+PNI: PRRSV+porcine-negative IgG; PRRSV+ICs: PRRSV+porcine IgG against PRRSV; RNI: rabbit-negative IgG; pIRF3: phosphorylated interferon regulatory factor 3; GAPDH: glyceraldehyde-3-phosphate dehydrogenase; FcγRI: Fc gamma receptor I; FcγRIII: Fc gamma receptor III.

As shown in Figure 2a, IFN-α mRNA level in PRRSV-infected cells was significantly upregulated at 12 and 24 h post-infection and weakly downregulated at 36–72 h post-infection, compared to mock cells. IFN-α protein level in culture supernatants of PRRSV-infected cells was significantly more than that in culture supernatants of mock cells at 12–60 h post-infection, showing a peak at 24 h post-infection and rapidly decreasing thereafter (Figure 3a). As shown in Figure 2b, TNF-α mRNA level in PRRSV-infected cells was significantly upregulated at any time point post-infection, compared to mock cells. However, TNF-α protein level in culture supernatants of PRRSV-infected cells was slightly more than that in culture supernatants of mock cells at any time point post-infection (Figure 3b). As shown in Figure 2c and Figure 3c, both the IL-10 mRNA level in PRRSV-infected cells and its protein level in culture supernatants of PRRSV-infected cells were significantly upregulated at any time point post-infection, compared to mock cells. As shown in Figure 4a, the phosphorylated IFN regulatory factor 3 (pIRF3) was detected in poly (I:C)-treated cells at any time point post-treatment (lane 5) and in PRRSV-infected cells at 12 and 24 h post-infection (lane 2), but undetected in PRRSV-infected cells at 36–72 h post-infection (lane 2).

### 3.3. PRRSV-ADE Infection DownRegulates the Production of IFN-α and TNF-α in Porcine AMs

As shown in Figure 2a and Figure 3a, both the IFN-α mRNA level in PRRSV+ICs-infected cells and its protein level in culture supernatants of PRRSV+ICs-infected cells were significantly downregulated at any time point post-infection, compared to PRRSV+PNI-infected cells. As shown in Figure 2b and Figure 3b, both the TNF-α mRNA level in PRRSV+ICs-infected cells and its protein level in culture supernatants of PRRSV+ICs-infected cells were also significantly downregulated at any time point post-infection, compared to PRRSV+PNI-infected cells. However, both the IL-10 mRNA level in PRRSV+ICs-infected cells and its protein level in culture supernatants of PRRSV+ICs-infected cells were significantly upregulated at any time point post-infection, compared to PRRSV+PNI-infected cells (Figure 2c and Figure 3c). As shown in Figure 4a, the amount of pIRF3 in PRRSV+ICs-infected cells (lane 4) was visibly less than that in PRRSV+PNI-infected cells (lane 3) at 12 and 24 h post-infection.

### 3.4. FcγRI and FcγRIII in Porcine AMs Are Involved in PRRSV-ADE Infection

**Figure 5:**
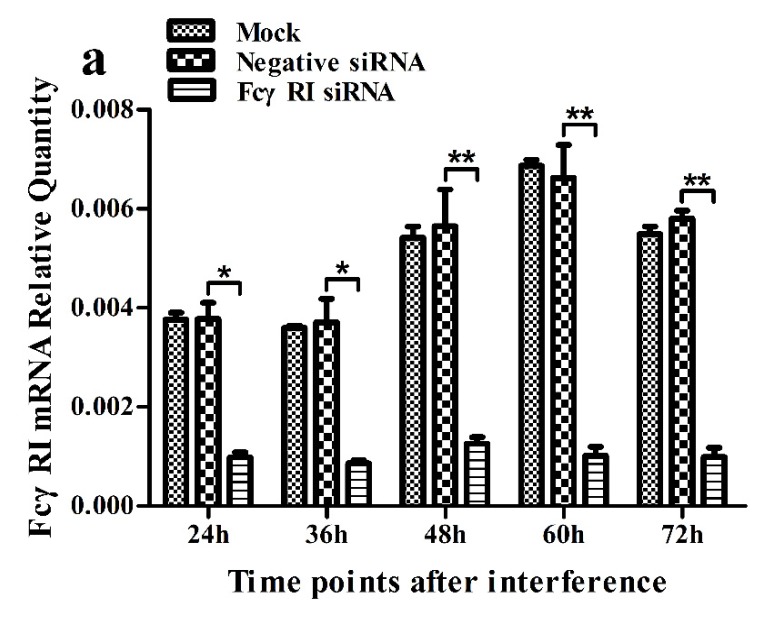

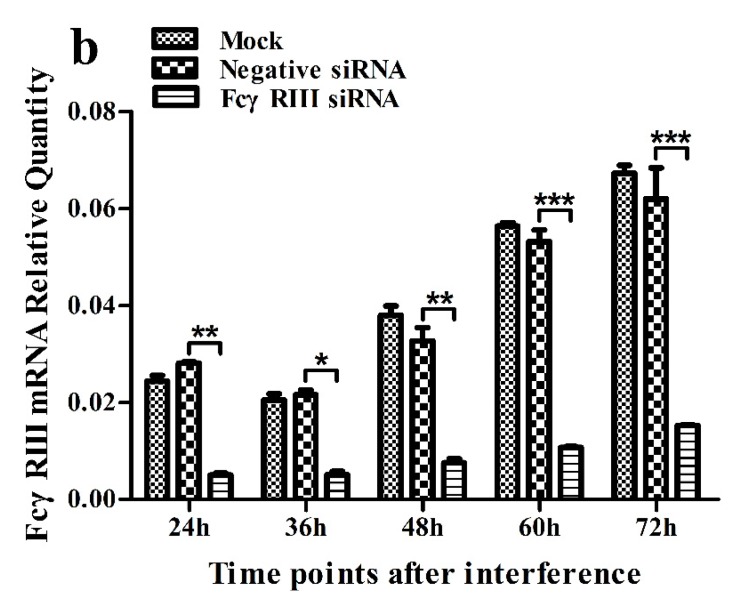
Relative quantitative RT-PCR analysis of mRNA expression of FcγRI or FcγRIII in porcine AMs. (**a**) Relative quantitative RT-PCR analysis of FcγRI mRNA level in cells transfected with FcγRI siRNA or negative siRNA. (**b**) Relative quantitative RT-PCR analysis of FcγRIII mRNA level in cells transfected with FcγRIII siRNA or negative siRNA. *** *p* < 0.001, ** *p* < 0.01, * *p* < 0.05. Note: siRNA: small interfering RNA; FcγRI: Fc gamma receptor I; FcγRIII: Fc gamma receptor III.

**Figure 6:**
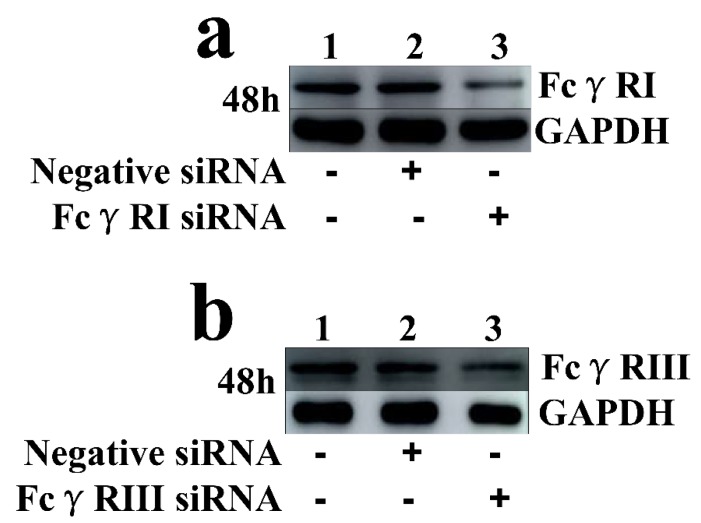
Immunoblot analysis of protein expression of FcγRI or FcγRIII in porcine AMs. (**a**) FcγRI protein level in cells transfected with FcγRI siRNA or negative siRNA for 48 h was quantified bywestern blot. (**b**) FcγRIII protein level in cells transfected with FcγRIII siRNA or negative siRNA for 48 h was quantified by western blot. The GAPDH served as a loading control. Note: siRNA: small interfering RNA; FcγRI: Fc gamma receptor I; FcγRIII: Fc gamma receptor III; GAPDH: glyceraldehyde-3-phosphate dehydrogenase.

**Figure 7:**
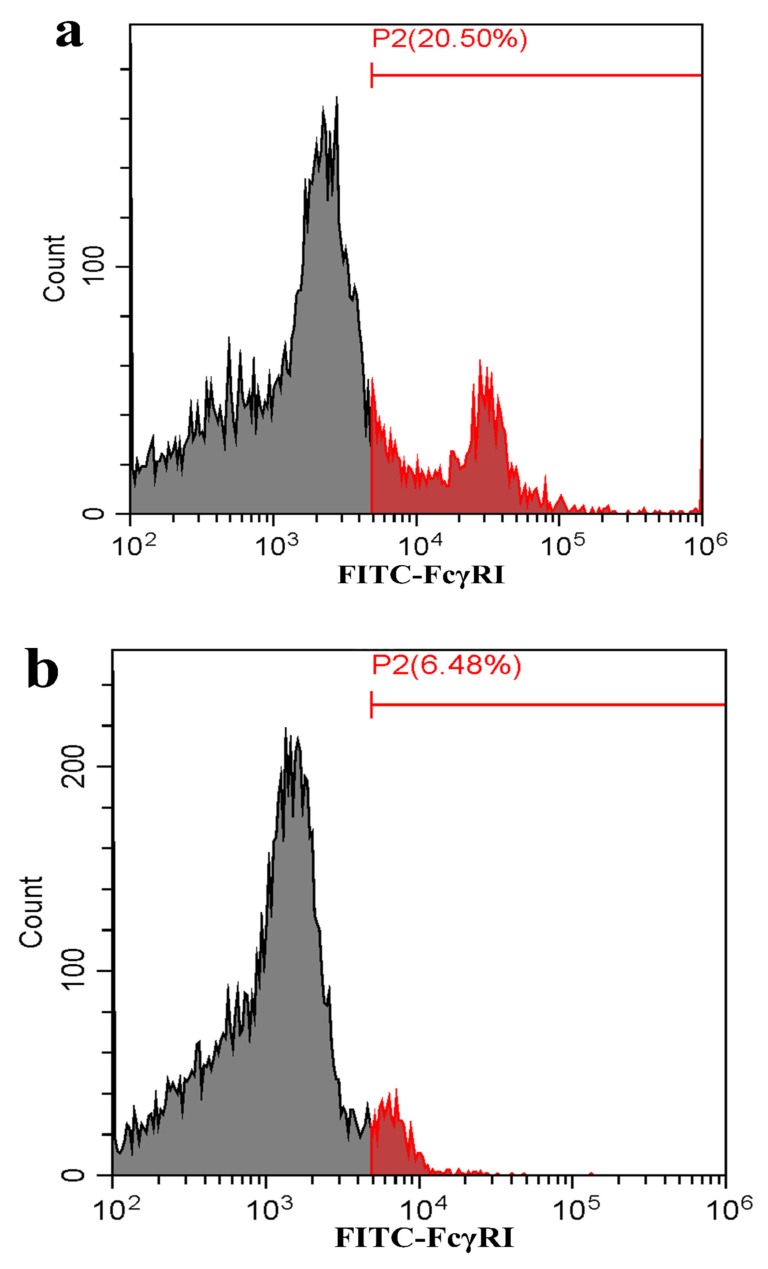

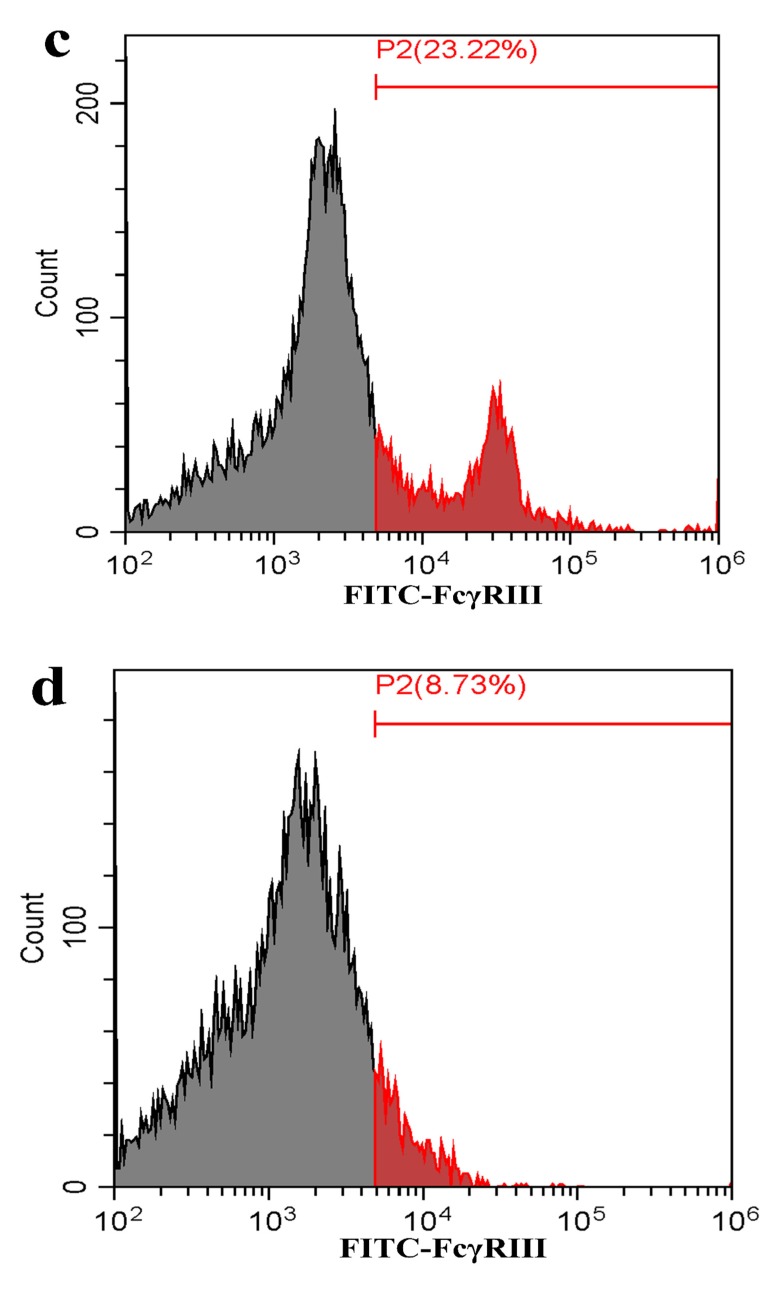

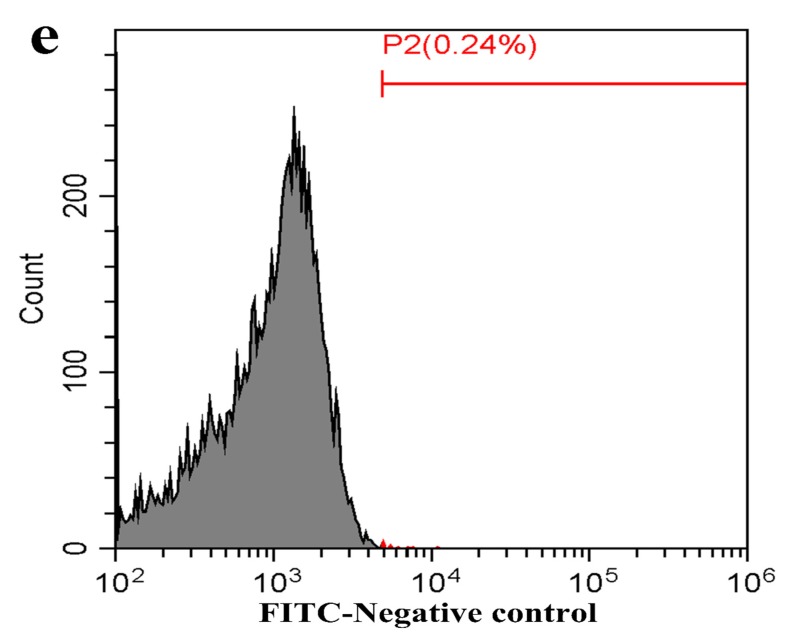
Flow cytometry analysis of FcγRI or FcγRIII on the surface of porcine AMs. The cells were transfected with FcγRI siRNA, FcγRIII siRNA or negative siRNA for 48 h. (**a**) Representative histograms of the percentage of negative siRNA-transfected cells surface-stained with anti-FcγRI IgG and FITC-conjugated goat anti-rabbit IgG. (**b**) Representative histograms of the percentage of FcγRI siRNA-transfected cells surface-stained with anti-FcγRI IgG and FITC-conjugated goat anti-rabbit IgG. (**c**) Representative histograms of the percentage of negative siRNA-transfected cells surface-stained with anti-FcγRIII IgG and FITC-conjugated goat anti-rabbit IgG. (**d**) Representative histograms of the percentage of FcγRIII siRNA-transfected cells surface-stained with anti-FcγRIII IgG and FITC-conjugated goat anti-rabbit IgG. (**e**) Representative histograms of the percentage of mock cells surface-stained with RNI and FITC-conjugated goat anti-rabbit IgG. Note: siRNA: small interfering RNA; FcγRI: Fc gamma receptor I; FcγRIII: Fc gamma receptor III; FITC: fluorescein isothiocyanate; RNI: rabbit-negative IgG.

**Figure 8:**
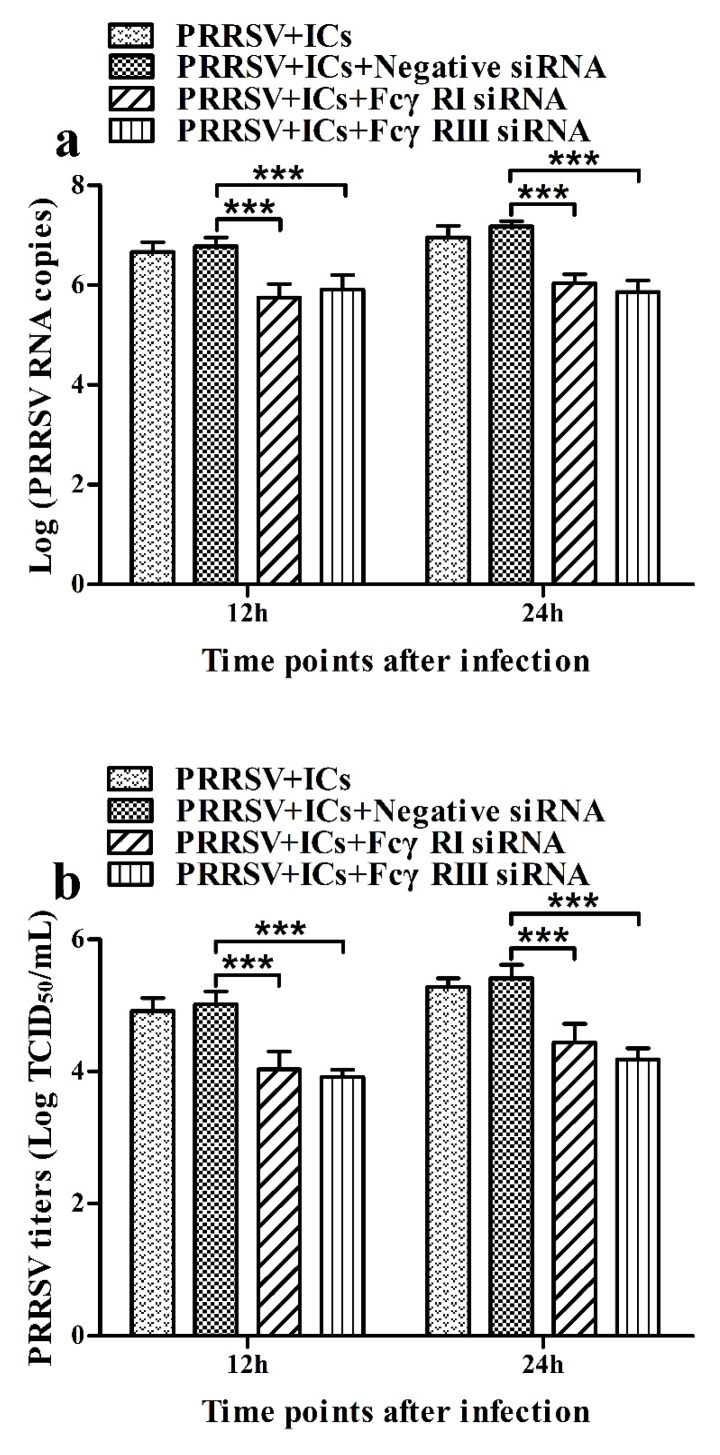
Effect of FcγRI gene knockdown or FcγRIII gene knockdown on PRRSV replication by the ADE infection in porcine AMs. The cells were transfected with FcγRI siRNA, FcγRIII siRNA or negative siRNA for 48h and then infected with PRRSV+ICs. (**a**) Real-time RT-PCR analysis of PRRSV RNA copies in culture supernatants of PRRSV+ICs-infected cells transfected with FcγRI siRNA, FcγRIII siRNA or negative siRNA. (**b**) PRRSV titers in culture supernatants of PRRSV+ICs-infected cells transfected with FcγRI siRNA, FcγRIII siRNA or negative siRNA from three independent experiments were measured in Marc-145 cells and expressed as TCID_50_/mL. *** *p* < 0.001. Note: siRNA: small interfering RNA; FcγRI: Fc gamma receptor I; FcγRIII: Fc gamma receptor III; ADE: antibody-dependent enhancement; PRRSV+ICs: PRRSV+porcine IgG against PRRSV.

As shown in Figure 5, both the FcγRI mRNA level in FcγRI siRNA-transfected cells and the FcγRIII mRNA level in FcγRIII siRNA-transfected cells were significantly downregulated at 24–72 h post-transfection, compared to negative siRNA-transfected cells. Simultaneously, both the FcγRI protein level in FcγRI siRNA-transfected cells and the FcγRIII protein level in FcγRIII siRNA-transfected cells were visibly reduced at 48 h post-transfection, compared to negative siRNA-transfected cells (Figure 6). Additionally, both FcγRI on the surface of FcγRI siRNA-transfected cells and FcγRIII on the surface of FcγRIII siRNA-transfected cells were also reduced at 48 h post-transfection, compared to negative siRNA-transfected cells (Figure 7). After 48 h transfection, the transfected cells were infected with PRRSV+ICs. As shown in Figure 8, both PRRSV RNA copies and its titers in culture supernatants of PRRSV+ICs-infected cells transfected with FcγRI siRNA or FcγRIII siRNA were significantly less than that in culture supernatants of PRRSV+ICs-infected cells transfected with negative siRNA at 12 and 24 h post-infection.

### 3.5. Activation of FcγRI or FcγRIII Down-Regulates the Production of IFN-α and TNF-α in Porcine AMs

**Figure 9:**
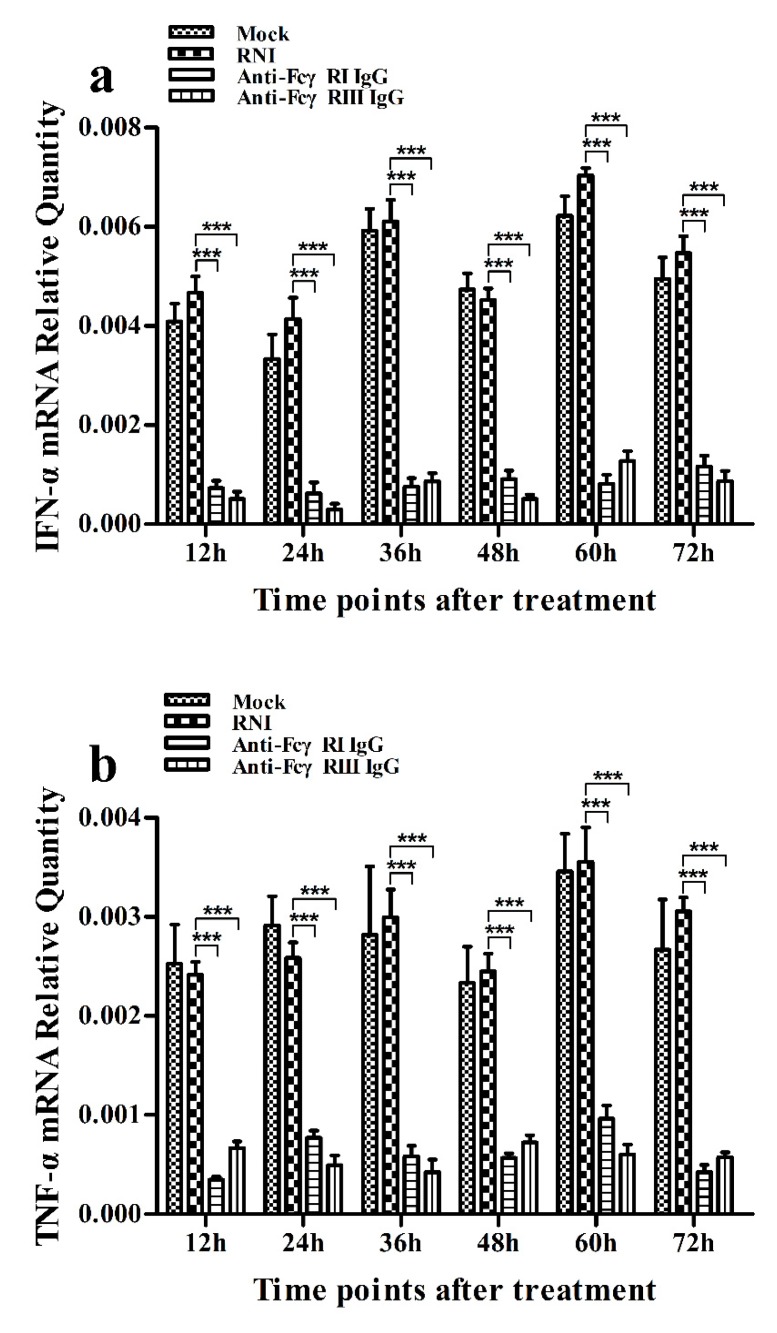

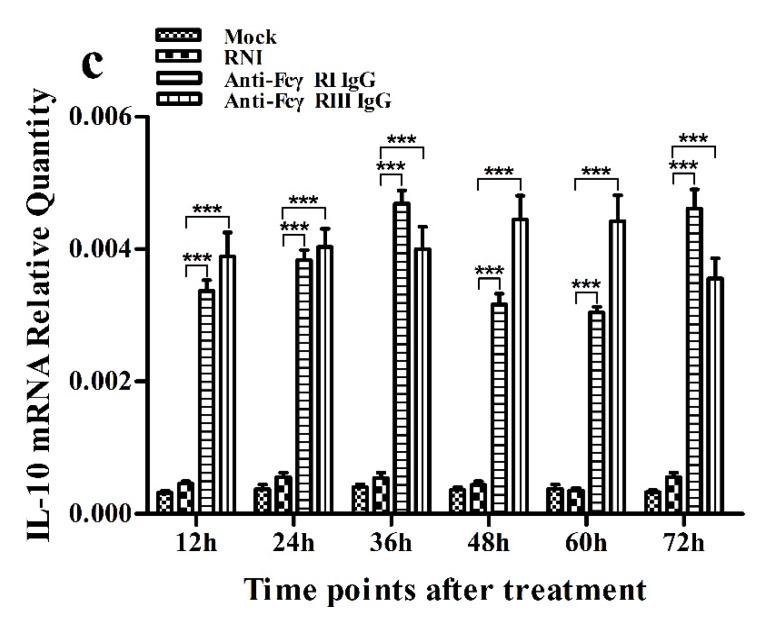
Effect of FcγRI or FcγRIII on mRNA expression of cytokines in porcine AMs. Relative quantitative RT-PCR analysis of cytokine mRNA levels in cells treated with RNI, anti-FcγRI IgG or anti-FcγRIII IgG. (**a**) IFN-α mRNA level, (**b**) TNF-α mRNA level, (**c**) IL-10 mRNA level. *** *p* < 0.001.

**Figure 10:**
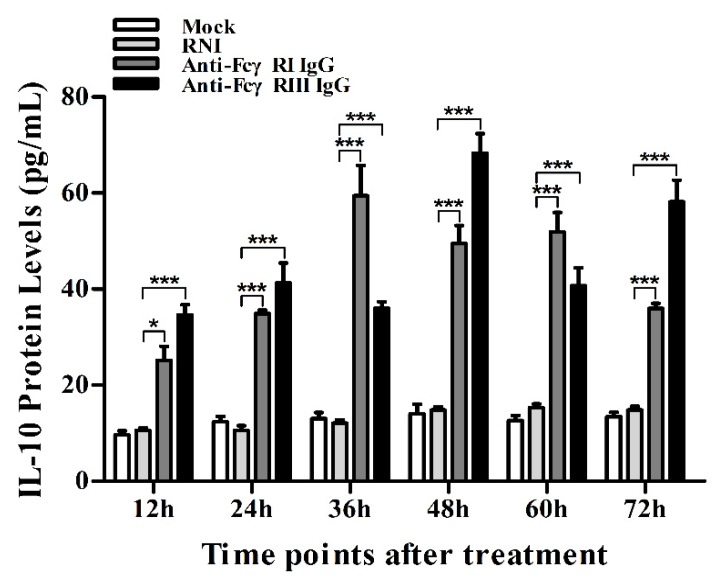
Effect of FcγRI or FcγRIII on protein expression of IL-10 in porcine AMs. IL-10 protein level in culture supernatants of cells treated with RNI, anti-FcγRI IgG or anti-FcγRIII IgG was detected by using the IL-10 ELISA Kit. *** *p* < 0.001, * *p* < 0.05.

As shown in Figure 9a,b, both IFN-α and TNF-α mRNA levels in cells treated with anti-FcγRI IgG or anti-FcγRIII IgG were significantly downregulated at any time point post-treatment, compared to cells treated with RNI. Simultaneously, examination of culture supernatants of cells treated with anti-FcγRI IgG or anti-FcγRIII IgG yielded negative results for IFN-α and TNF-α. However, both the IL-10 mRNA level in cells treated with anti-FcγRI IgG or anti-FcγRIII IgG and its protein level in culture supernatants of cells treated with anti-FcγRI IgG or anti-FcγRIII IgG were significantly upregulated at any time point post-treatment, compared to cells treated with RNI (Figure 9c and Figure 10). As shown in Figure 4b, the pIRF3 was undetected in cells treated with anti-FcγRI IgG or anti-FcγRIII IgG at any time point post-treatment (lane 3,4).

### 3.6. Activation of FcγRI or FcγRIII Downregulates Poly (I:C)-Induced Production of IFN-α and TNF-α in Porcine AMs

**Figure 11:**
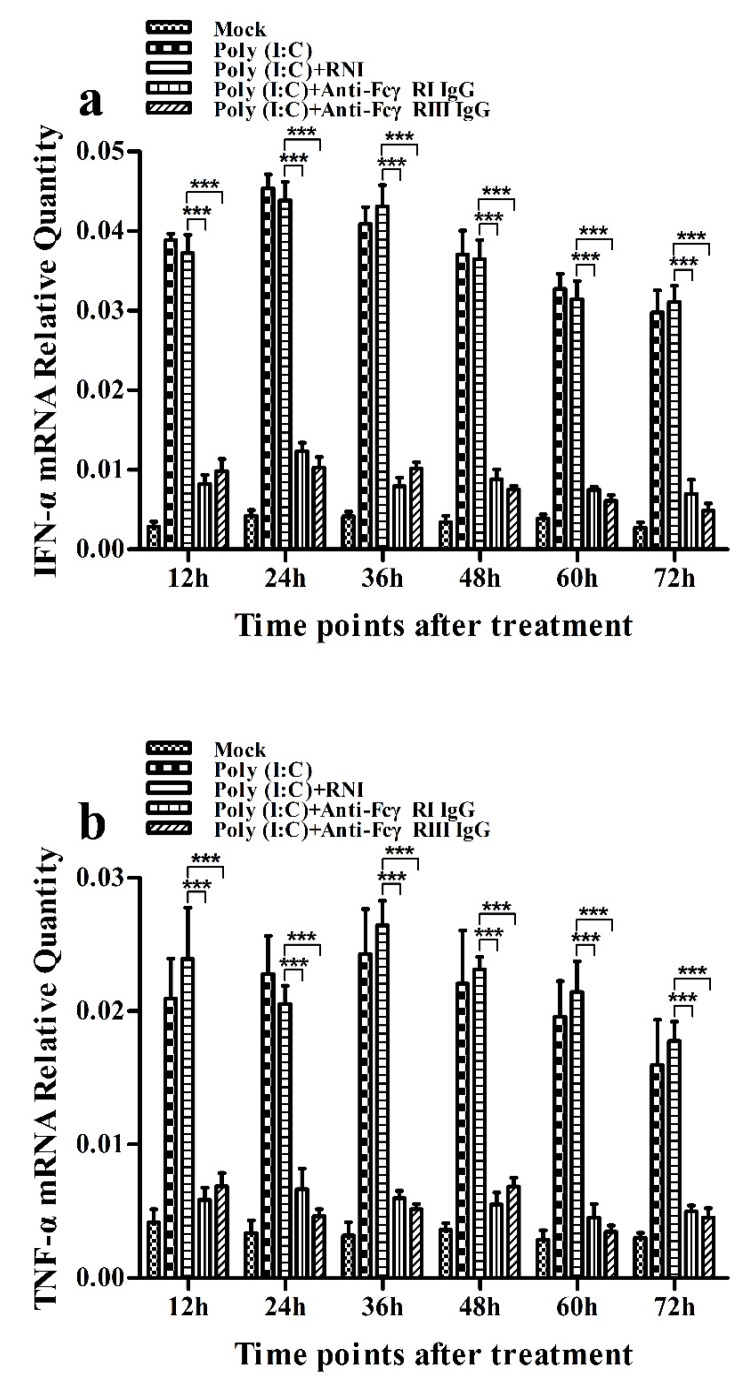

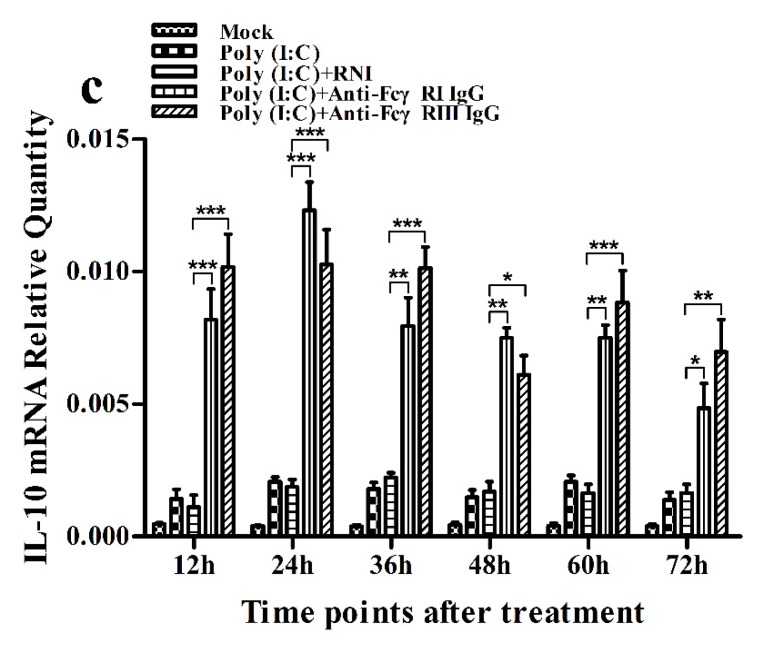
Effect of FcγRI or FcγRIII on poly (I:C)-induced mRNA expression of cytokines in porcine AMs. Relative quantitative RT-PCR analysis of cytokine mRNA levels in poly (I:C)-stimulated cells pretreated with RNI, anti-FcγRI IgG or anti-FcγRIII IgG. (**a**) IFN-α mRNAl evel, (**b**) TNF-α mRNA level, (**c**) IL-10 mRNA level. *** *p* < 0.001, ** *p* < 0.01, * *p* < 0.05.

**Figure 12:**
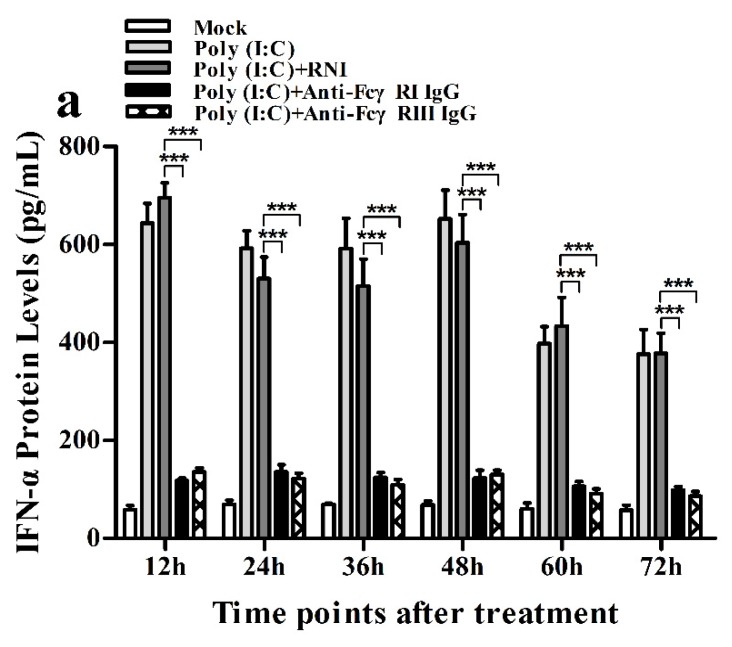

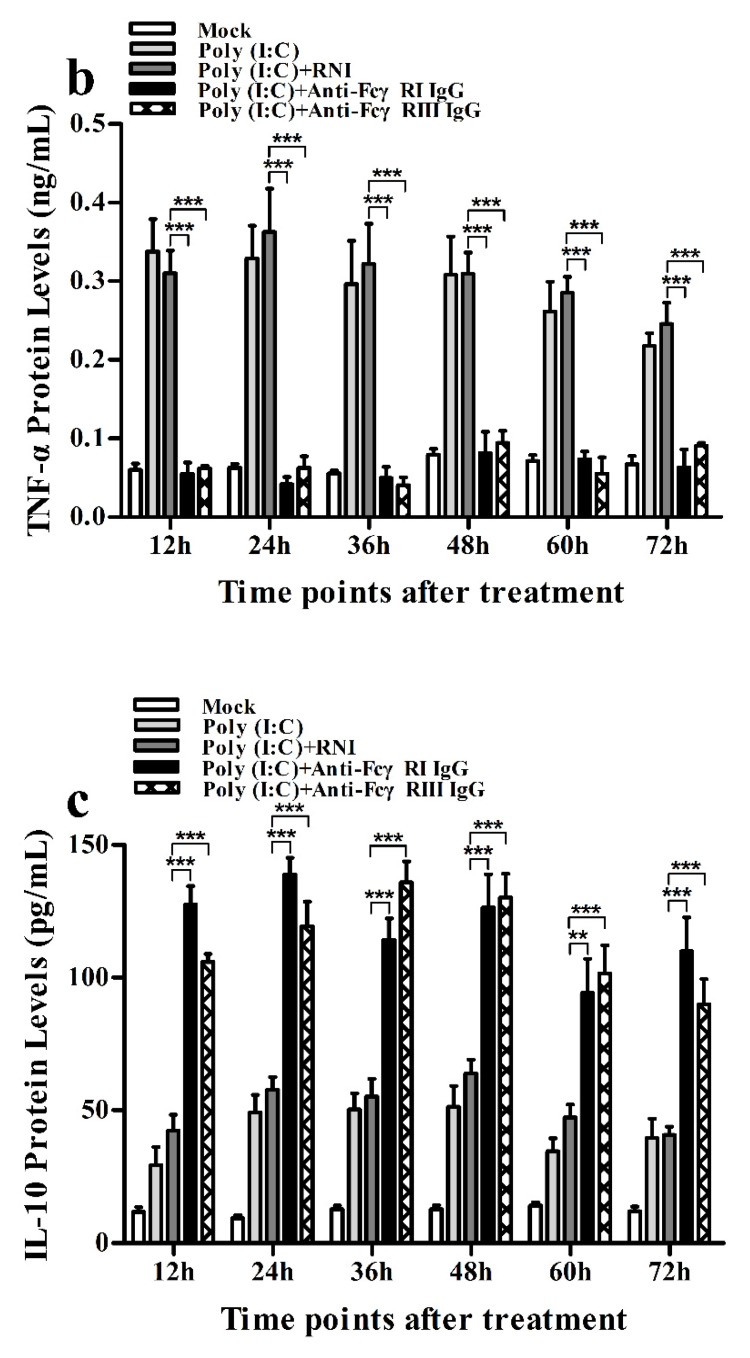
Effect of FcγRI or FcγRIII on poly (I:C)-induced protein expression of cytokines in porcine AMs. Cytokine protein levels in culture supernatants of poly (I:C)-stimulated cells pretreated with RNI, anti-FcγRI IgG or anti-FcγRIII IgG were detected by using ELISA Kits. (**a**) IFN-α protein level, (**b**) TNF-α protein level, (**c**) IL-10 protein level. *** *p* < 0.001, ** *p* < 0.01.

As shown in Figure 11a and Figure 12a, both the IFN-α mRNA level in poly (I:C)-stimulated cells pretreated with anti-FcγRI IgG or anti-FcγRIII IgG and its protein level in culture supernatants of poly (I:C)-stimulated cells pretreated with anti-FcγRI IgG or anti-FcγRIII IgG were significantly downregulated at any time point post-treatment, compared to poly (I:C)-stimulated cells pretreated with RNI. As shown in Figure 11b and Figure 12b, both the TNF-α mRNA level in poly (I:C)-stimulated cells pretreated with anti-FcγRI IgG or anti-FcγRIII IgG and its protein level in culture supernatants of poly (I:C)-stimulated cells pretreated with anti-FcγRI IgG or anti-FcγRIII IgG were also significantly downregulated at any time point post-treatment, compared to poly (I:C)-stimulated cells pretreated with RNI. However, both the IL-10 mRNA level in poly (I:C)-stimulated cells pretreated with anti-FcγRI IgG or anti-FcγRIII IgG and its protein level in culture supernatants of poly (I:C)-stimulated cells pretreated with anti-FcγRI IgG or anti-FcγRIII IgG were significantly upregulated at any time point post-treatment, compared to poly (I:C)-stimulated cells pretreated with RNI (Figure 11c and Figure 12c). As shown in Figure 4c, the amount of pIRF3 in poly (I:C)-stimulated cells pretreated with anti-FcγRI IgG or anti-FcγRIII IgG (lane 4,5) was obviously less than that in poly (I:C)-stimulated cells pretreated with RNI (lane 3) at any time point post-treatment.

### 3.7. Activation of FcγRI or FcγRIII Downregulates PRRSV-Induced Production of IFN-α and TNF-α in Porcine AMs

**Figure 13:**
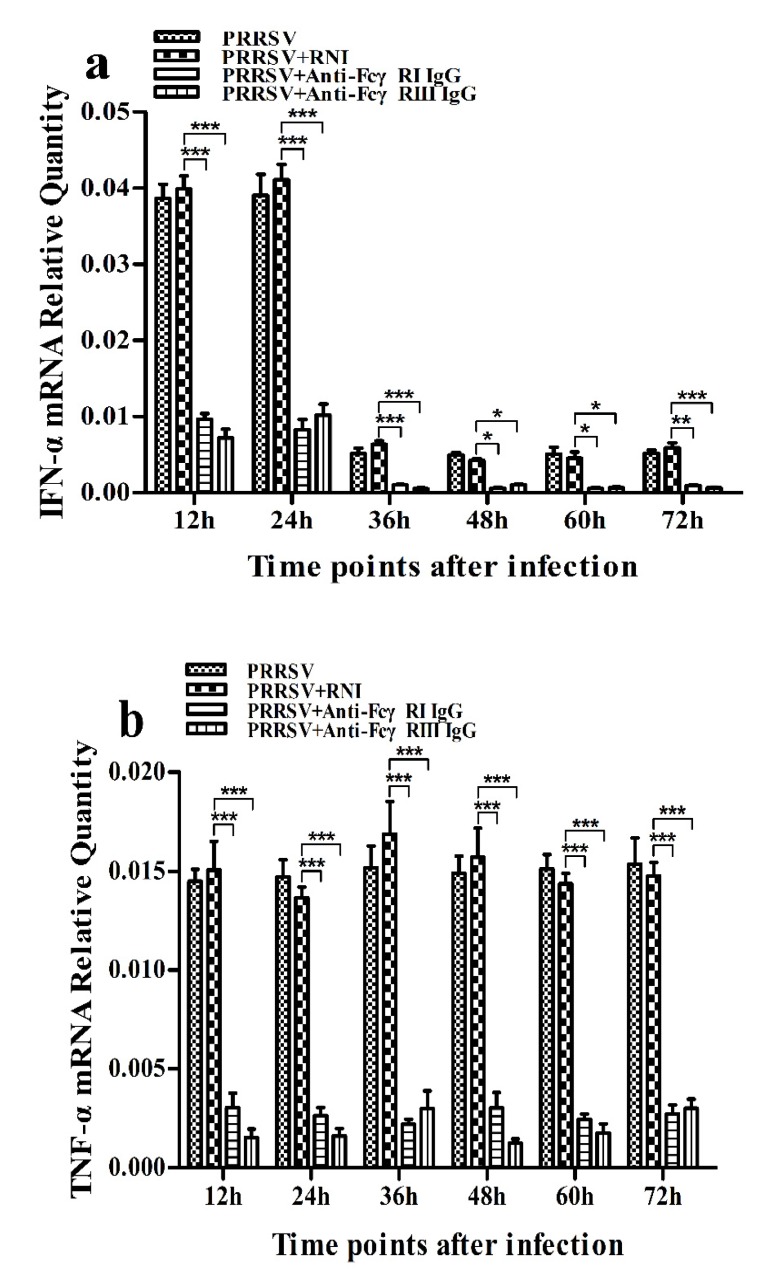

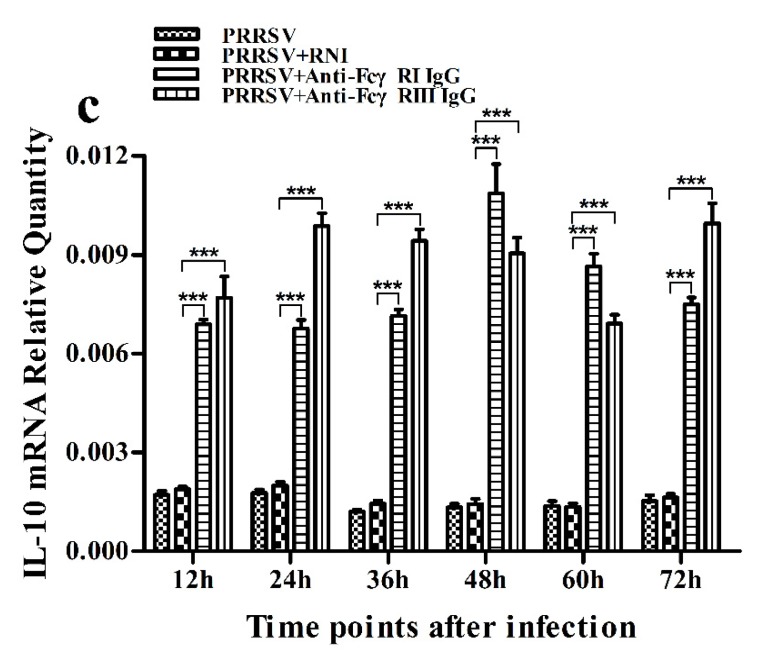
Effect of FcγRI or FcγRIII on PRRSV-induced mRNA expression of cytokines in porcine AMs. Relative quantitative RT-PCR analysis of cytokine mRNA levels in PRRSV-infected cells pretreated with RNI, anti-FcγRI IgG or anti-FcγRIII IgG. (**a**) IFN-α mRNA level, (**b**) TNF-α mRNA level, (**c**) IL-10 mRNA level. *** *p* < 0.001, ** *p* < 0.01, * *p* < 0.05.

**Figure 14:**
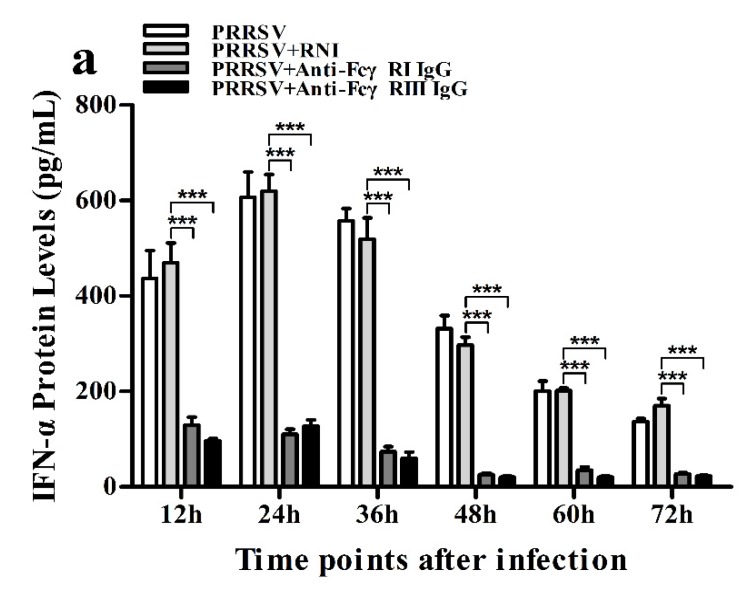

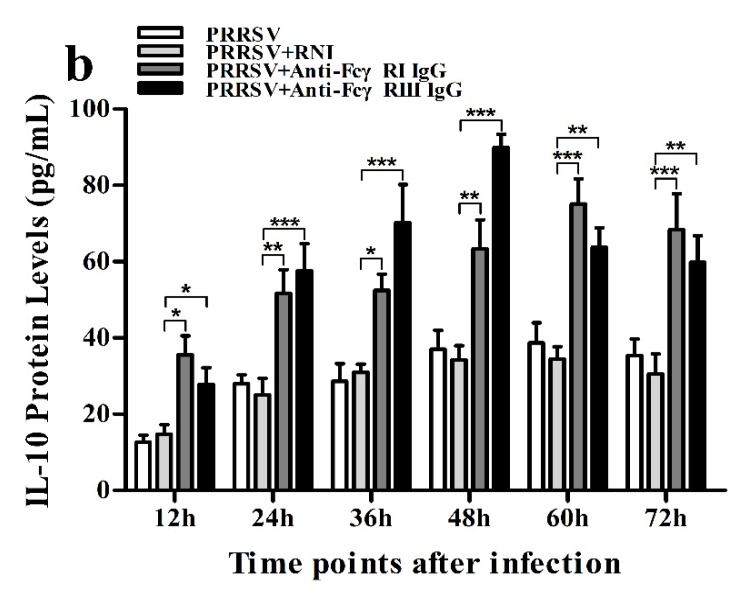
Effect of FcγRI or FcγRIII on PRRSV-induced protein expression of cytokines in porcine AMs. Cytokine protein levels in culture supernatants of PRRSV-infected cells pretreated with RNI, anti-FcγRI IgG or anti-FcγRIII IgG were detected by using ELISA Kits. (**a**) IFN-α protein level, (**b**) IL-10 protein level. *** *p* < 0.001, ** *p* < 0.01, * *p* < 0.05.

As shown in Figure 13a and Figure 14a, both the IFN-α mRNA level in PRRSV-infected cells pretreated with anti-FcγRI IgG or anti-FcγRIII IgG and its protein level in culture supernatants of PRRSV-infected cells pretreated with anti-FcγRI IgG or anti-FcγRIII IgG were significantly downregulated at any time point post-infection, compared to PRRSV-infected-cells pretreated with RNI. As shown in Figure 13b, the TNF-α mRNA level in PRRSV-infected cells pretreated with anti-FcγRI IgG or anti-FcγRIII IgG was significantly downregulated at any time point post-infection, compared to PRRSV-infected-cells pretreated with RNI. Simultaneously, examination of culture supernatants of PRRSV-infected cells pretreated with anti-FcγRI IgG or anti-FcγRIII IgG yielded a negative result for TNF-α. However, both the IL-10 mRNA level in PRRSV-infected cells pretreated with anti-FcγRI IgG or anti-FcγRIII IgG and its protein level in culture supernatants of PRRSV-infected cells pretreated with anti-FcγRI IgG or anti-FcγRIII IgG were significantly upregulated at any time point post-infection, compared to PRRSV-infected-cells pretreated with RNI (Figure 13c and Figure 14b). As shown in Figure 4d, the amount of pIRF3 in PRRSV-infected cells pretreated with anti-FcγRI IgG or anti-FcγRIII IgG (lane 4,5) was visibly less than that in PRRSV-infected cells pretreated with RNI (lane 3) at 12 and 24 h post-infection.

### 3.8. Activation of FcγRI or FcγRIII Facilitates PRRSV Replication in Porcine AMs

**Figure 15:**
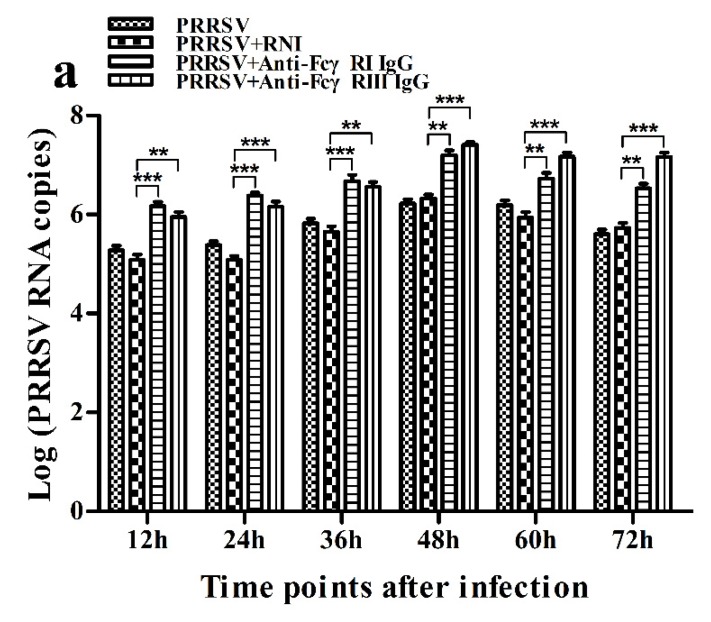

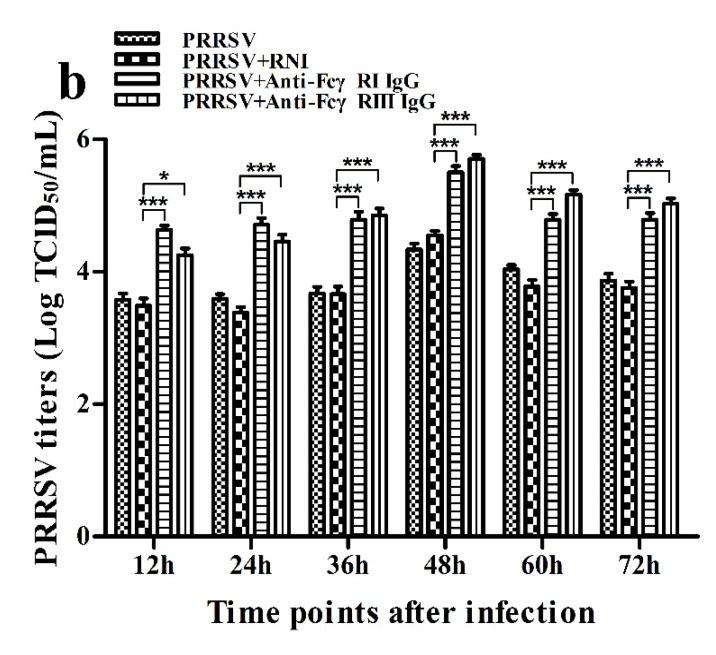
Effect of FcγRI or FcγRIII on PRRSV replication in porcine AMs. (**a**) Real-time RT-PCR analysis of PRRSV RNA copies in culture supernatants of PRRSV-infected cells pretreated with RNI, anti-FcγRI IgG or anti-FcγRIII IgG. (**b**) PRRSV titers in culture supernatants of PRRSV-infected cells pretreated with RNI, anti-FcγRI IgG or anti-FcγRIII IgG from three independent experiments were measured in Marc-145 cells and expressed as TCID_50_/mL. *** *p* < 0.001, ** *p* < 0.01, * *p* < 0.05.

As shown in Figure 15, both PRRSV RNA copies and its titers in culture supernatants of PRRSV-infected cells pretreated with anti-FcγRI IgG or anti-FcγRIII IgG were significantly more than that in culture supernatants of PRRSV-infected cells pretreated with RNI at any time point post-infection.

## 4. Discussion

Type I IFNs (IFN-α and IFN-β) play crucial roles in the innate immune system defense against viral infection by inducing an antiviral immune response [32]. PRRSV infection usually induces an inefficient or absent host immune response with the inhibition of IFN-α production [31,33,34]. Nevertheless, different PRRSV field isolates may differ in IFN-α induction [35]. Our studies showed that PRRSV induced a high level of IFN-α production in porcine AMs in early infection and slightly inhibited IFN-α production in porcine AMs in late infection, which was in accordance with previous studies [35,36,37]. Meanwhile, the mRNA levels of interferon-stimulated gene 15 (ISG15), ISG56 and 2′, 5′-oligoadenylate synthetase (OAS2) in PRRSV-infected porcine AMs were significantly more than that in mock porcine AMs (Supplementary Figure S1). These results suggest that PRRSV infection induced an IFN-α antiviral response in host cells. IRF3 is a critical transcription factor in initiating type I IFN production [38]. The phosphorylation of Ser^396^ within the Serine/Threonine (Ser/Thr) cluster of IRF3 is essential for IRF3 activation [39]. We tested if PRRSV infection resulted in IRF3 phosphorylation in porcine AMs by using poly (I:C) as a positive control. We observed that the pIRF3 was detected in poly (I:C)-stimulated cells at 12–72 h post-treatment and in PRRSV-infected cells at 12 and 24 h post-infection, but undetectable in PRRSV-infected cells at 36–72 h post-infection. Thus, it was possible that PRRSV induced the production of IFN-α in porcine AMs, maybe by activating IRF3 phosphorylation at the early phase of infection. TNF-α is a pleiotropic cytokine mainly secreted by monocytes and macrophages. In addition to its direct antiviral effect, TNF-α also induces and regulates the inflammatory response [40]. IL-10 is important in viral infection, which may relate to immunosuppression of the hosts by inhibiting the synthesis of proinflammatory cytokines [41]. PRRSV seems to induce chaotic TNF-α and IL-10 responses. The available evidence has shown that different PRRSV isolates induce different patterns of TNF-α and IL-10 [42,43,44]. Our studies showed that PRRSV infection induced the production of TNF-α and IL-10 in porcine AMs.

FcγR is an important bridge between the innate and adaptive immune systems. However, FcγR’s role in antiviral immune response to viral infection has not yet been studied extensively. An early study has shown that FcγRI in macrophages from mice promotes the production of IL-10 and inhibits the secretion of IL-12 [45]. Subsequent another study showed that the ligation of FcγR in murine bone marrow-derived macrophages essentially abrogates lipopolysaccharide-induced production of IL-12 by dramatically increasing the production of IL-10, but TNF-α production is not affected by the FcγR ligation [46]. A recent study shows that FcγRIIa inhibits type I and III IFN production by human myeloid immune cells [47]. Our studies showed that activation of FcγRI or FcγRIII in porcine AMs downregulated the production of IFN-α and TNF-α, and upregulated the production of IL-10. Our studies also showed that activation of FcγRI or FcγRIII in porcine AMs downregulated poly (I:C)-induced production of IFN-α and TNF-α, and upregulated poly (I:C)-induced production of IL-10. These results suggested that porcine activating FcγR signaling suppressed the innate antiviral immune response. Immunoblot analysis showed that activation of FcγRI or FcγRIII reduced poly (I:C)-induced phosphorylation of IRF3 in porcine AMs, suggesting that porcine activating FcγR signaling inhibited poly (I:C)-induced production of IFN-α, maybe by blocking poly (I:C)-induced phosphorylation of IRF3. Furthermore, our studies showed that activation of FcγRI or FcγRIII in porcine AMs downregulated PRRSV-induced production of IFN-α and TNF-α, but upregulated PRRSV-induced production of IL-10 and facilitated PRRSV replication. Simultaneously, PRRSV-induced mRNA levels of ISG15, ISG56 and OAS2 in porcine AMs were downregulated by the activation of FcγRI or FcγRIII (Supplementary Figure S2). These results suggested that porcine activating FcγR signaling suppressed the innate antiviral immune response to PRRSV infection.

Early studies have shown that ADE of the dengue virus infection downregulates the production of IFN-β, IFN-γ, TNF-α and IL-12, and upregulates the production of IL-6 and IL-10 [48,49,50]. The downregulation of IFN-β and TNF-α expression is also observed in ADE of ross river virus infection or PRRSV infection [51,52]. But, the exact mechanisms by which ADE of virus infection inhibits the production of innate antiviral cytokines are unknown. Our studies showed that the production of IFN-α and TNF-α in PRRSV+ICs-infected porcine AMs was significantly less than that in PRRSV+PNI-infected porcine AMs, whereas the production of IL-10 in PRRSV+ICs-infected porcine AMs was significantly more than that in PRRSV+PNI-infected porcine AMs. Meanwhile, the mRNA levels of ISG15, ISG56 and OAS2 in PRRSV+ICs-infected porcine AMs were significantly less than that in PRRSV+PNI-infected porcine AMs (Supplementary Figure S1). These results suggested that PRRSV-ADE infection suppressed the innate antiviral immune response by inhibiting the production of IFN-α and TNF-α. To determine whether porcine activating FcγRs influenced PRRSV-ADE infection, porcine AMs were transfected with FcγRIsiRNA, FcγRIIIsiRNA or negative siRNA for 48 h and then infected with PRRSV+ICs. Our studies showed that the knockdown of FcγRI or FcγRIII in porcine AMs significantly suppressed ADE of PRRSV infection, suggesting that both porcine FcγRI and FcγRIII were involved in PRRSV-ADE infection, which was similar to recent studies [26,53]. Simultaneously, the knockdown of FcγRI or FcγRIII in porcine AMs during PRRSV-ADE infection significantly upregulated the production of IFN-α and TNF-α, while it significantly downregulated the production of IL-10 (Figures S3 and S4). Moreover, our studies showed that both porcine FcγRI and FcγRIII signaling suppressed the innate antiviral immune response to PRRSV infection by inhibiting the production of IFN-α and TNF-α in porcine AMs. Taken together, our studies suggested that PRRSV-ADE infection inhibited the production of innate antiviral cytokines (IFN-α and TNF-α), maybe by porcine activating FcγRs (FcγRI and FcγRIII), hence facilitating viral replication.

## Figures and Tables

**Table 1:** Small interfering RNAs (siRNAs) used in this study.

Target Gene Name ^#^	Sequence (5′-3′)
FcγRI	Sense: GCCUUGAGGUGUCAUGGAUTT Antisense: AUCCAUGACACCUCAAGGCTT
FcγRIII	Sense: GUGGAGAAUACACGUGUAATT Antisense: UUACACGUGUAUUCUCCACTT
Negative control	Sense: UUCUCCGAACGUGUCACGUTT Antisense: ACGUGACACGUUCGGAGAATT

^#^ FcγRI: Fc gamma receptor I; FcγRIII: Fc gamma receptor III.

**Table 2:** Relative quantitative real-time polymerase chain reaction (RT-PCR) primers used in this study.

Primer Name ^#^	Primer Sequence (5′-3′)	
IFN-α F	GGATCAGCAGCTCAGGG	
IFN-α R	GAGGGTGAGTCTGTGGAAGTA	
TNF-α F	AGCCGCATCGCCGTCTCCTAC	
TNF-α R	CCTGCCCAGATTCAGCAAAGTCC	
IL-10 F	GCATCCACTTCCCAACCA	
IL-10 R	TCGGCATTACGTCTTCCAG	
ISG15 F	GGTGCAAAGCTTCAGAGACC	[31]
ISG15 R	GTCAGCCAGACCTCATAGGC	
ISG56 F	TCAGAGGTGAGAAGGCTGGT	[31]
ISG56 R	GCTTCCTGCAAGTGTCCTTC	
OAS2 F	CACAGCTCAGGGATTTCAGA	[31]
OAS2 R	TCCAACGACAGGGTTTGTAA	
FcγRI F	TGAAACAAAGTTGCTCCCA	
FcγRI R	GCTGCGCTTGATGACCT	
FcγRIII F	CTGCTGCTTCTGGTTTCA	
FcγRIII R	CCATTCCACCTCCACTC	
β-actin F	CGGGACATCAAGGAGAAGC	
β-actin R	CTCGTTGCCGATGGTGATG	

^#^ F: forward primer; R: reverse primer; IFN-α: interferon-α; TNF-α: tumor necrosis factor-α; IL-10: interleukine-10; ISG15: interferon-stimulated gene 15; ISG56: interferon-stimulated gene 56; OAS2: 2′, 5′-oligoadenylate synthetase; FcγRI: Fc gamma receptor I; FcγRIII: Fc gamma receptor III.

## Supplementary Materials

The following are available online at https://www.mdpi.com/1999-4915/12/2/187/s1, Figure S1: Effect of PRRSV infection or PRRSV-ADE infection on mRNA expression of antiviral genes in porcine AMs, Figure S2: Effect of FcγRI or FcγRIII on PRRSV-induced mRNA expression of antiviral genes in porcine AMs, Figure S3: Effect of FcγRI gene knockdown or FcγRIII gene knockdown on mRNA expression of cytokines in porcine AMs during PRRSV-ADE infection, Figure S4: Effect of FcγRI gene knockdown or FcγRIII gene knockdown on protein expression of cytokines in porcine AMs during PRRSV-ADE infection.
