# Supplementary material for: Antibody-Mediated Porcine Reproductive and Respiratory Syndrome Virus Infection Downregulates the Production of Interferon-α and Tumor Necrosis Factor-α in Porcine Alveolar Macrophages via Fc Gamma Receptor I and III
Source: Viruses. 2020 Feb 8;12(2):187. doi: 10.3390/v12020187 (PMC7077232; doi:10.3390/v12020187)
Supplement: Supplementary file 1 [file viruses-12-00187-s001.pdf]

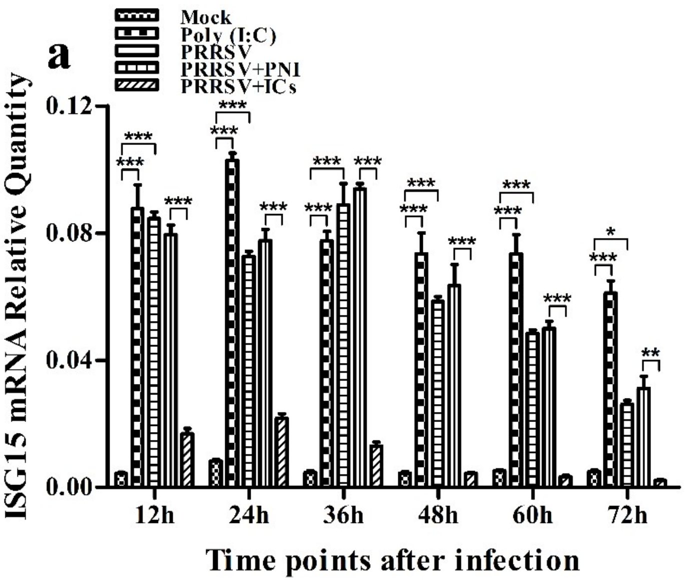

a
Mock
Poly (I:C)
PRRSV
PRRSV+PNI
PRRSV+ICs
ISG15 mRNA Relative Quantity
0.12
0.08
0.04
0.00
12h
24h
36h
48h
60h
72h
Time points after infection

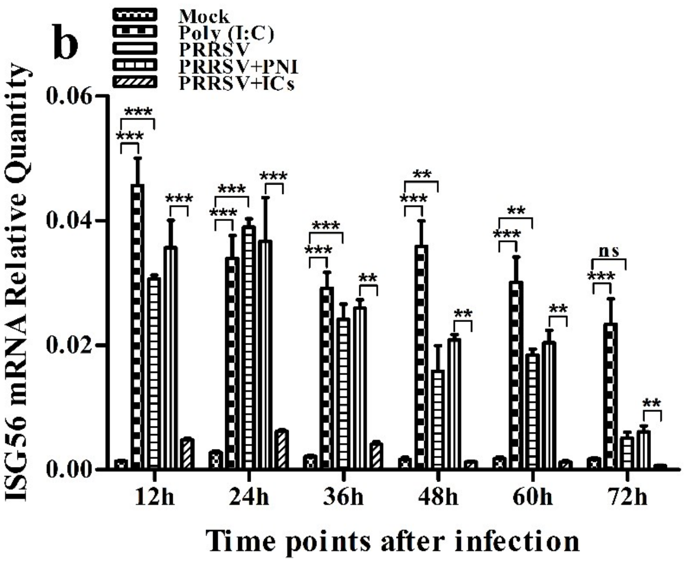

b
Mock
Poly (I:C)
PRRSV
PRRSV+PNI
PRRSV+ICs
ISG56 mRNA Relative Quantity
0.06
0.04
0.02
0.00
12h
24h
36h
48h
60h
72h
Time points after infection

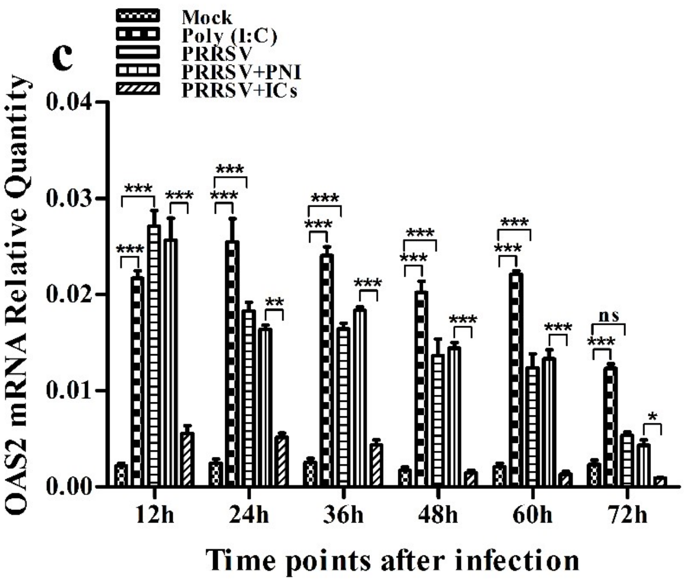

Figure S1. Effect of PRRSV infection or PRRSV-ADE infection on mRNA expression of antiviral genes in porcine AMs. Relative quantitative RT-PCR analysis of antiviral gene mRNA levels in mock cells or poly (I:C)-stimulated cells or PRRSV-infected cells or PRRSV+PNI-infected cells or PRRSV+ICs-infected cells. (a) ISG15 mRNA level; (b) ISG56 mRNA level; (c) OAS2 mRNA level. ***p< 0.001, **p< 0.01, *p< 0.05, ns: no significance.

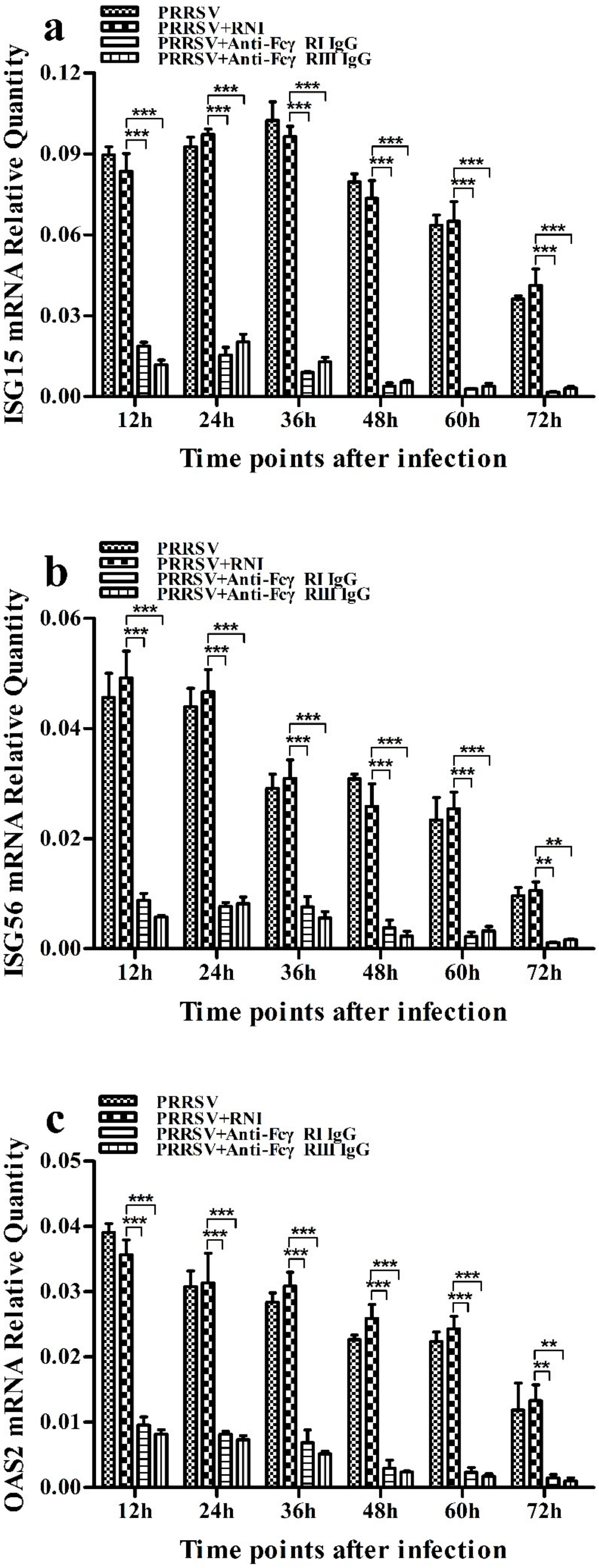
a
PRRSV
PRRSV+RNI
PRRSV+Anti-Fcγ RI IgG
PRRSV+Anti-Fcγ RIII IgG
ISG15 mRNA Relative Quantity
0.12
0.09
0.06
0.03
0.00
12h
24h
36h
48h
60h
72h
Time points after infection
b
PRRSV
PRRSV+RNI
PRRSV+Anti-Fcγ RI IgG
PRRSV+Anti-Fcγ RIII IgG
ISG56 mRNA Relative Quantity
0.06
0.04
0.02
0.00
12h
24h
36h
48h
60h
72h
Time points after infection
c
PRRSV
PRRSV+RNI
PRRSV+Anti-Fcγ RI IgG
PRRSV+Anti-Fcγ RIII IgG
OAS2 mRNA Relative Quantity
0.05
0.04
0.03
0.02
0.01
0.00
12h
24h
36h
48h
60h
72h
Time points after infection

Figure S2. Effect of FcγRI or FcγRIII on PRRSV-induced mRNA expression of antiviral genes in porcine AMs. Relative quantitative RT-PCR analysis of antiviral gene mRNA levels in PRRSV-infected cells pretreated with RNI or anti-FcγRI IgG or anti-FcγRIII IgG. (a) ISG15 mRNA level; (b) ISG56 mRNA level; (c) OAS2 mRNA level. ***$p < 0.001$, **$p < 0.01$.

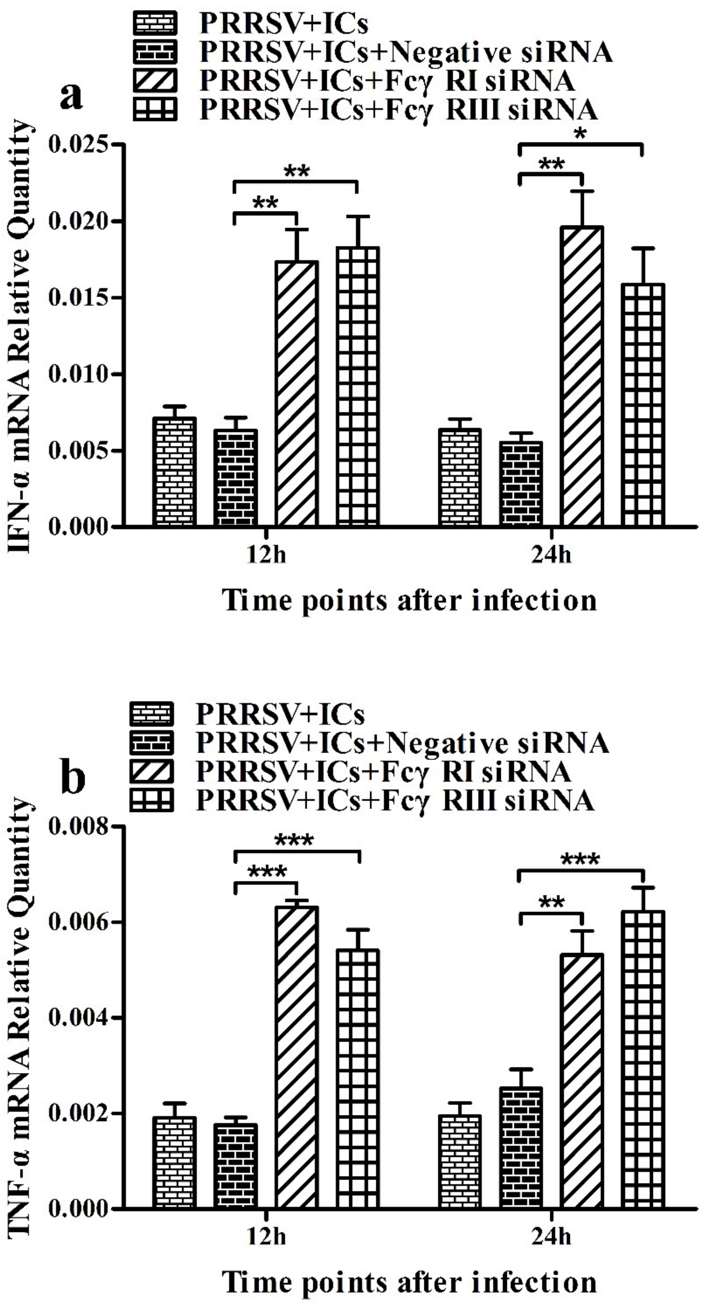
a
PRRSV+ICs
PRRSV+ICs+Negative siRNA
PRRSV+ICs+Fcγ RI siRNA
PRRSV+ICs+Fcγ RIII siRNA
IFN-α mRNA Relative Quantity
0.025
0.020
0.015
0.010
0.005
0.000
**
**
**
*
12h
24h
Time points after infection
b
PRRSV+ICs
PRRSV+ICs+Negative siRNA
PRRSV+ICs+Fcγ RI siRNA
PRRSV+ICs+Fcγ RIII siRNA
TNF-α mRNA Relative Quantity
0.008
0.006
0.004
0.002
0.000
***
***
***
**
12h
24h
Time points after infection

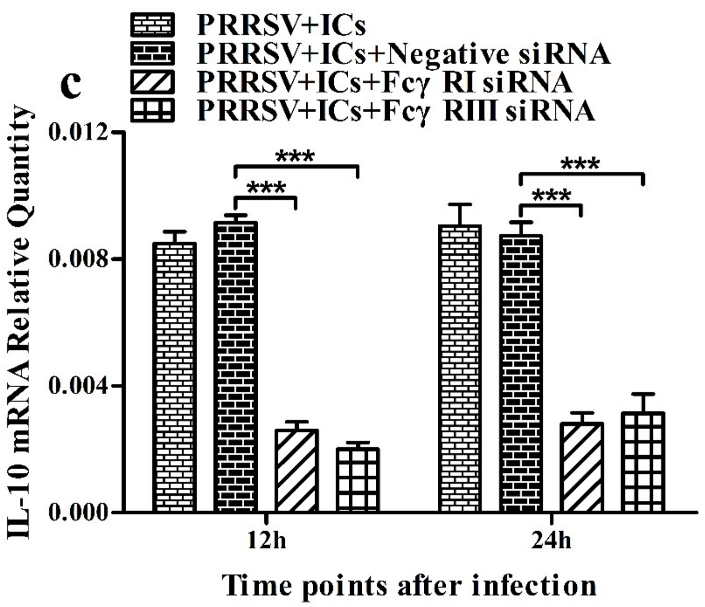

Figure S3. Effect of FcγRI gene knockdown or FcγRIII gene knockdown on mRNA expression of cytokines in porcine AMs during PRRSV-ADE infection. The cells were transfected with FcγRI siRNA or FcγRIII siRNA or negative siRNA for 48h and then infected with PRRSV+ICs. Relative quantitative RT-PCR analysis of cytokine mRNA levels in PRRSV+ICs-infected cells transfected with FcγRI siRNA or FcγRIII siRNA or negative siRNA. (a) IFN-α mRNA level; (b) TNF-α mRNA level; (c) IL-10 mRNA level. ***$p< 0.001$, **$p< 0.01$, *$p< 0.05$.

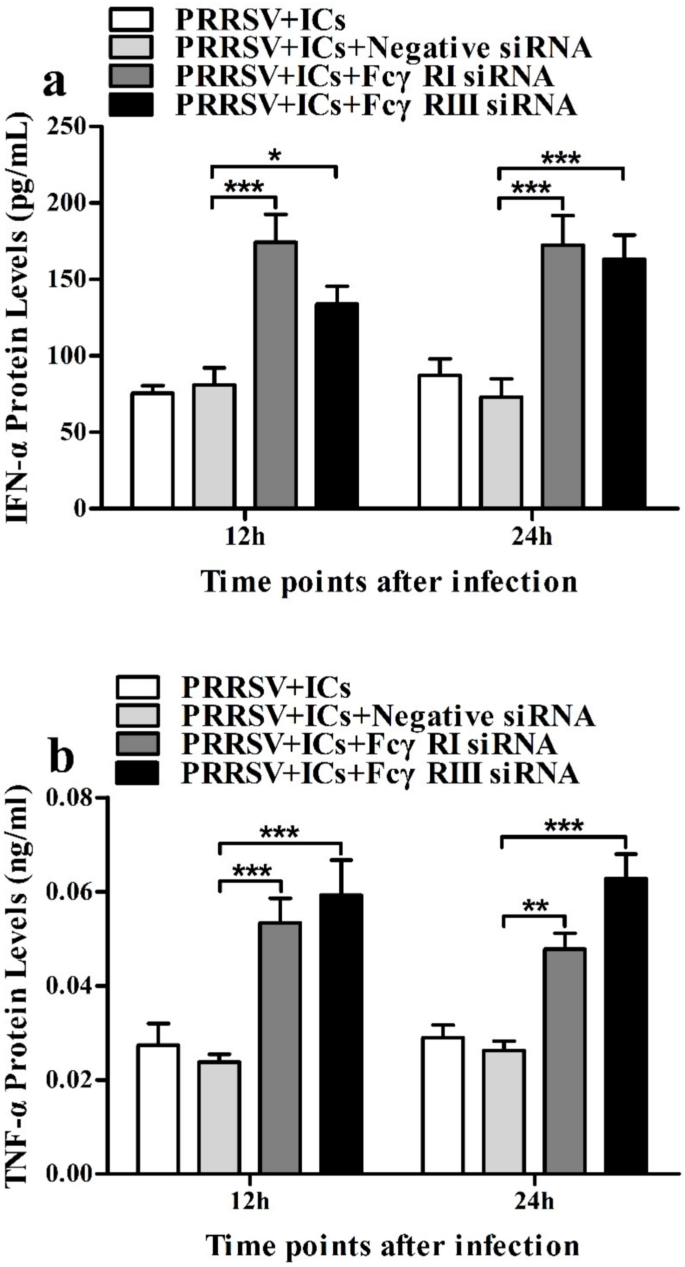
a
PRRSV+ICs
PRRSV+ICs+Negative siRNA
PRRSV+ICs+Fcγ RI siRNA
PRRSV+ICs+Fcγ RIII siRNA
IFN-α Protein Levels (pg/mL)
250
200
150
100
50
0
*
***
***
***
12h
24h
Time points after infection
b
PRRSV+ICs
PRRSV+ICs+Negative siRNA
PRRSV+ICs+Fcγ RI siRNA
PRRSV+ICs+Fcγ RIII siRNA
TNF-α Protein Levels (ng/ml)
0.08
0.06
0.04
0.02
0.00
***
***
***
**
12h
24h
Time points after infection

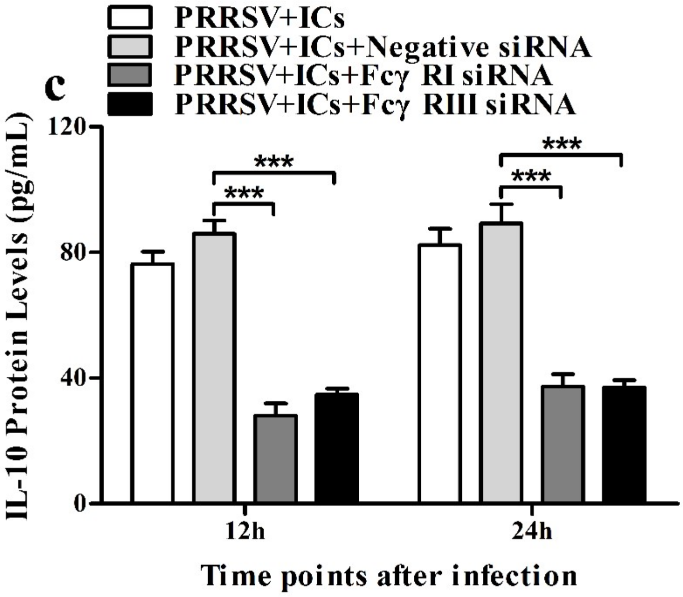

Figure S4. Effect of FcγRI gene knockdown or FcγRIII gene knockdown on protein expression of cytokines in porcine AMs during PRRSV-ADE infection. The cells were transfected with FcγRI siRNA or FcγRIII siRNA or negative siRNA for 48h and then infected with PRRSV+ICs. Cytokine protein levels in culture supernatants of PRRSV+ICs-infected cells transfected with FcγRI siRNA or FcγRIII siRNA or negative siRNA were detected by using ELISA Kits. (a) IFN-α protein level; (b) TNF-α protein level; (c) IL-10 protein level. ***$p < 0.001$, **$p < 0.01$, *$p < 0.05$.
